# Efficient Synthesis of the Fungal Phytotoxin (±)-3-Deoxyradicinin

**DOI:** 10.3390/toxins18100412

**Published:** 2026-09-24

**Authors:** Marilena Caporale, Giulia Marsico, Patrizia Scafato, Stefano Superchi

**Affiliations:** Department of Basic and Applied Sciences, University of Basilicata, Via dell’Ateneo Lucano 10, 85100 Potenza, Italy; marilena.caporale@unibas.it (M.C.); giuliamar86.gm@gmail.com (G.M.)

**Keywords:** fungal phytotoxins, α-pyrones, total synthesis, process optimization, gram-scale synthesis, bioherbicides

## Abstract

An efficient synthesis of phytotoxin (±)-3-deoxyradicinin was developed from commercially available 4-hydroxy-6-methyl-2H-pyran-2-one. The propenyl side chain was introduced through regioselective bromination followed by Horner–Wadsworth–Emmons olefination, while the second pyranone ring was constructed via a Lewis acid-catalyzed reaction with crotonic acid. The synthetic route was designed according to the principles of operational simplicity, with the aim of minimizing the use of hazardous reagents and solvents, as well as demanding purification procedures and cryogenic conditions. These objectives were successfully achieved for most of the synthetic sequence, with the use of CCl_4_ in the bromination step representing the main remaining limitation. Four of the six steps were successfully performed on a gram scale, demonstrating the practical potential of the route for a possible scale-up. In addition, a practical synthesis of the natural product 5,6-dehydrokawain was established. Overall, 319 mg of (±)-3-deoxyradicinin were obtained in 23% overall yield, providing a practical approach with reduced reliance on hazardous reagents and low-temperature conditions. This synthetic strategy therefore represents a useful platform for further scale-up of 3-deoxyradicinin preparation and for the synthesis of structurally related analogues.

## 1. Introduction

Fungal phytotoxins are low-molecular-weight secondary metabolites produced by plant-pathogenic fungi that play a central role in the pathogenesis of numerous economically significant crop diseases [1,2]. They constitute a structurally diverse class of natural products, encompassing a wide range of polycyclic and heterocyclic scaffolds, many of which display potent biological activities toward host plants and other organisms. The isolation and characterization of fungal phytotoxins have therefore been a longstanding focus of natural products chemistry, driven not only by their relevance to plant pathology and agriculture but also by their potential applications in biotechnology, pharmaceutical research, and agrochemical development. In agriculture, particular attention has been devoted to their potential use as selective herbicides and biocontrol agents [3,4,5]. The inherent selectivity of many host-selective toxins toward particular plant species makes them attractive candidates for the development of natural herbicides that may pose fewer environmental and health risks than conventional synthetic herbicides. Beyond their agricultural applications, many phytotoxins exhibit potent activity in mammalian or microbial assay systems, suggesting potential pharmaceutical uses [6,7]. Indeed, some host-selective toxins have been reported to display cytotoxic activity against mammalian cancer cell lines or antimicrobial activity against clinically relevant pathogens. The structural complexity and potent bioactivity of many fungal phytotoxins have made them attractive targets for synthetic and biosynthetic investigations. Furthermore, the development of efficient synthetic routes to these compounds facilitates both mechanistic studies on their biological activity and the creation of structural analogs with potentially improved properties for agricultural or pharmaceutical applications. Although fermentation-based production of phytotoxins is theoretically feasible and may be economically favorable for large-scale synthesis, several challenges limit its practical application [1,8]. These include the variable and often low titers achieved in liquid culture, the self-toxicity of some metabolites toward the producing organism, metabolic instability under standard fermentation conditions, and difficulties in achieving consistent product quality and purity. These limitations have motivated extensive research into the chemical synthesis of phytotoxins and their structural analogs. In this framework, radicinin (**1**) (Figure 1) can be considered one of the most promising fungal phytotoxins for both agricultural and pharmaceutical applications. It was first isolated in 1953 from the fungus *Stemphylium radicinum* [9], and later from many other pathogenic fungi [10,11,12,13,14,15,16]. The structure of radicinin was determined in 1964 by Grove et al. [17] whereas its absolute configuration was later inferred by ECD spectroscopy [11] and supported by X-ray crystallographic analysis of the corresponding 4-*O*-*p*-bromobenzoyl ester [10]. In the agricultural field, radicinin (**1**) has demonstrated high potential as a target-specific bioherbicide against buffelgrass (*Cenchrus ciliaris*), a highly invasive perennial grass-weed in North America and Australia [18], as well as phytotoxic activity against the radicle growth of *Amaranthus retroflexus* [19], antifungal activity [20], and antibacterial activity against *Xylella fastidiosa*, the causal agent of many devastating plant diseases [21]. Notably, radicinin (**1**) demonstrated high target-specific toxicity on buffelgrass, low toxicity to native plants, and no teratogenic, sub-lethal, or lethal effects on zebrafish (*Brachydanio rerio*) embryos [18]. The potential of **1** for pharmaceutical applications is also noteworthy, as it exhibits antibiotic activity against Gram-positive bacteria [19,20], antiviral activity [14], and anticancer activity against human cancer cell lines derived from non-small-cell lung carcinoma, oligodendroglioma, and melanoma [22]. We recently reported a novel synthesis of racemic 3-deoxyradicinin (**2**) (Figure 1) [23], the immediate biosynthetic precursor of **1** [24]. Compound **2** displayed activity comparable to that of naturally occurring radicinin (**1**), both as a herbicide against buffelgrass [23] and as an anticancer agent [22]. Moreover, (±)-**2** and **1** showed comparable inhibitory activity against the Gram-negative bacteria *X. fastidiosa* and *Liberibacter crescens* [25]. Notably, the enantioenriched (−)-**2** and (+)-**2** samples also exhibited comparable bactericidal activity, suggesting that the mechanism of action of this compound is not stereospecific [25]. These findings indicate that neither the hydroxy group at C-3 of **1** nor the absolute configuration of the methyl-substituted stereogenic center at C-2 plays a major role in determining its biological activity. Therefore, (±)-3-deoxyradicinin (**2**), which is considerably easier to prepare than optically active radicinin (**1**), represents a practical alternative to the natural phytotoxin and is thus a valuable synthetic target. Indeed, the use of racemic mixtures or achiral compounds instead of enantiopure chiral substances offers considerable advantages when producing the large quantities required for extensive field trials in agriculture and/or preclinical studies in medicine.

Although several syntheses of racemic 3-deoxyradicinin (**2**) have been reported, a truly efficient, safe, and sustainable route suitable for large-scale production is still lacking. Existing procedures typically involve a large number of steps, low overall yields, and the use of difficult to handle organometallic reagents, making them unsuitable for scale-up [23,24,25,26] (vide infra). This limitation highlights the need for new, cost-effective synthetic strategies that comply with green chemistry principles and can be potentially translated into industrial applications. Therefore, we undertook a study aimed at defining a practical synthetic route to (±)-3-deoxyradicinin (**2**) which could, in principle, be amenable to scale-up. Our goal was then to develop a procedure which avoids dangerous and/or toxic reagents, limits the use of solvents, employs easy purification steps, and provides a good overall yield. A further goal was to design a strategy also versatile for the synthesis of analogues of **2** bearing different side-chains at C-7.

## 2. Results and Discussion

To achieve the targets outlined in the Introduction, we designed the synthetic route to (±)-3-deoxyradicinin (**2**) depicted in Scheme 1, in which the non-toxic and inexpensive commercially available 4-hydroxy-6-methyl-2*H*-pyran-2-one (**3**), already containing the α-pyrone core of **2**, was selected as the starting material. Notably, several steps in this route were performed under mild conditions, often avoiding the use of solvents and costly purification procedures. Moreover, four of the six steps were successfully carried out on a gram scale, demonstrating the scalability and practical feasibility of the proposed route for future scale-up.

### 2.1. Optimization of Alkenyl Pyrone ***7*** Synthesis

Initially, the acidic hydroxyl group of **3** was protected by methylation to prevent interference during subsequent transformations. The methylation was performed using the mild base K_2_CO_3_ and trimethyl phosphate [27], a well-established benign methylating agent [28,29], affording the methyl-protected compound **4** in 90% yield. This procedure offers several notable advantages because the high solubility of starting material **3** in trimethyl phosphate eliminates the need for additional solvents. Moreover, the use of hazardous conventional methylating agents, such as dimethyl sulfate or methyl iodide, is avoided. Product isolation and purification were also straightforward and were achieved through precipitation with water, filtration, and crystallization from ethanol.

The introduction of the peripheral propenyl chain was initially attempted through two different approaches. First, direct access to the unsaturated compound **7** from α-pyrone **4** was investigated. This approach involved deprotonation of the lateral methyl group of **4**, followed by nucleophilic addition to acetaldehyde. The resulting aldol product was then subjected to dehydration to afford the desired unsaturated compound (Scheme 2). Several reaction conditions were screened using different bases, including Mg(OMe)_2_, NaHMDS, MeONa, *t*-BuONa, as well as various solvents (Table S1 in the Supplementary Materials), but no formation of product **7** was detected under the conditions reported in entries 1–3, and the starting material was quantitatively recovered. In contrast, under the conditions described in entry 4, product **7** was obtained in trace amounts, indicating that these conditions were only marginally effective in promoting the desired transformation. The failure of these attempts was attributed to the propensity of acetaldehyde to undergo self-condensation under the basic reaction conditions. Evidence supporting this hypothesis was obtained by employing non-enolizable aromatic benzaldehyde in reactions with the carbanion of methyl ether **4**. Treatment of methyl ether **4** with benzaldehyde in the presence of *t*-BuONa in DMF at room temperature (Table S1, entries 5 and 6) afforded 5,6-dehydrokawain (**9**) in 57% yield [30] (Scheme 2), a naturally occurring kavalactone isolated from the roots of kava (*Piper methysticum*) as well as from other plant species, including *Alpinia zerumbet* [31,32]. The present procedure allows compound **9** to be obtained in only two steps, thus providing a more convenient approach than those previously reported [33,34].

Following the unsuccessful attempts to directly obtain olefin **7**, an alternative strategy based on Wittig olefination was investigated. Accordingly, compound **4** had to be converted into brominated intermediate **5**, a suitable substrate for the subsequent transformations. Initially, the development of a *green* bromination protocol for compound **4** was explored. To this end, *N*-bromosaccharin [35], a non-conventional and non-hazardous brominating agent reported to be effective in benzylic bromination reactions [36], was selected. However, under various reaction conditions (Table S2 in the Supplementary Materials, entries 1–5), ring bromination rather than allylic bromination occurred, consistently affording only regioisomer **5a**. Additional attempts to develop a greener bromination protocol employed tribromoisocyanuric acid (TBCA) as the halogenating agent [37] (Table S2 entries 6 and 7). However, no halogenated products were obtained, even when the reaction was performed in different solvents (Table S2 in the Supplementary Materials). These unsuccessful attempts prompted us to adopt a classical Wohl-Ziegler radical bromination approach. The use of *N*-bromosuccinimide (NBS) in carbon tetrachloride (CCl_4_) represents a well-established protocol for benzylic/allylic radical bromination and requires the presence of a radical initiator, such as visible-light irradiation, dibenzoyl peroxide, or a combination of both [38]. In our case radical initiation was achieved by irradiation with a 60 W visible-light lamp and the crude product was purified by column chromatography, affording bromopyrone **5** in 66% yield (Table S2 entry 10). A concentration-dependent effect was also observed: higher concentrations of substrate **4** resulted in lower ratio of compound **5**, accompanied by increased formation of the 3-bromo regioisomer **5a**. Notably, the highest relative proportion and isolated yield of compound **5** were obtained under the selected 0.04 M concentration, while the use of only a slight excess of NBS (1.1 equiv) significantly suppressed the formation of the methyl-dibrominated byproduct **5b**. Although this protocol was rather efficient in providing **5**, the use of toxic CCl_4_ severely limited its environmental compatibility and its potential for large-scale application. Therefore, we screened several safer and more environmentally friendly solvents for the bromination with NBS, carrying out the reaction in dimethyl carbonate, methyl acetate, acetonitrile, methyl *tert*-butyl ether, and water (Table S2, entries 12–16). However, in all cases bromination of **4** exclusively occurred at the 3-position of the ring instead of at the allylic methyl group, providing only **5a**. Even the attempt to carry out the bromination in solvent-free mechanochemical conditions, grinding both solid **4** and NBS in a mortar, was unsuccessful (Table S2, entry 17). Thus, the NBS/CCl_4_ bromination protocol was the only one affording bromopyrone **5**. Compound **5** was then converted into phosphonate **6** via a Michaelis-Arbuzov reaction performed under solvent-free conditions. The transformation was achieved simply by dissolving compound **5** in trimethyl phosphite, heating the mixture at 60 °C for 5 h. After chromatographic purification product **6** was recovered in 93% yield. Notably, traces of by-products **5a** and **5b**, present in the crude mixture of **5**, remained unreacted and were easily separated from **6** during the purification step. Phosphonate **6** was subsequently subjected to a Horner-Wadsworth-Emmons (HWE) reaction [39] with acetaldehyde (1.0 M in THF) to afford alkene **7** (Scheme 3). Several reaction conditions were screened to identify the optimal protocol (Table 1). In all cases, alkene **7** was obtained, albeit, depending on the reaction conditions employed, in varying yields and different *E*:*Z* ratio of the newly formed double bond.

Reactions performed at 0 °C using NaH in anhydrous THF or MeONa in DMF (entries 1 and 2) provided complete conversion within 1 h, affording alkene **7** in high yield. In both cases, a 77:23 *E*:*Z* ratio of the double bond was observed. The use of the sterically hindered base sodium bis(trimethylsilyl)amide (NaHMDS) (entry 3), performed at room temperature, resulted in a marked decrease in yield with only a marginal improvement in *E*:*Z* selectivity. The first two sets of conditions were then repeated at room temperature under otherwise identical conditions (entries 4 and 5), resulting in shorter reaction times, although yield and stereoselectivity differed substantially. NaH in THF (entry 4) afforded an excellent yield with only a slight increase in the *E*:*Z* ratio relative to entry 1. However, these conditions, employing pyrophoric NaH in the flammable THF, appeared quite problematic for a possible scale up. In contrast, safer MeONa in DMF (entry 5) provided excellent stereoselectivity (*E*:*Z* = 96:4) at the expense of a somewhat reduced, but still acceptable, 76% yield. Extending the reaction time with NaH at room temperature led to deterioration of both yield and *E* selectivity (entry 6). To improve the *E*:*Z* ratio of alkene **7** isomerization was attempted by dissolving the compound in toluene in the presence of molecular iodine and heating at 100 °C for 1 h. This treatment raised the *E*:*Z* ratio to 99:1, albeit reducing the yield to 60%. Therefore, conditions reported in entry 5 were chosen as optimal in terms of both yield and stereoselectivity. Notably, the subsequent transformation of **7** into (±)-3-deoxyradicinin (**2**) (vide infra) also led to complete isomerization of the alkene to the *E* configuration, as revealed by ^1^H-NMR analysis. This installation of the unsaturated side chain on the α-pyrone ring via the HWE approach, allowing the use of variously substituted aliphatic, olefinic, or aromatic aldehydes, also appears to be a versatile strategy for preparing derivatives of **2** bearing different substituents at C-7.

### 2.2. Scale-Up of Alkenyl Pyrone **7** Synthesis

To carry out a gram scale procedure to obtain pyrone **7**, 4.0 g of hydroxy pyrone **3** were methylated by heating at reflux in trimethyl phosphate, used as both a reagent and a solvent, in the presence of potassium carbonate. After quenching with cold water, the resulting precipitate was filtered and washed with cold ethanol, affording **4** in 90% yield. The latter was dissolved in carbon tetrachloride, treated with NBS and dibenzoyl peroxide heating at reflux. After quenching and treatments, bromopyrone **5** was recovered together with small impurities of **5a** and **5b**. Crude **5** was then dissolved in trimethyl phosphite, used as both a reagent and a solvent, and heated at 60 °C for 5 h. After filtration of the crude on a silica plug, compound **6** was obtained in 60% overall yield over the last two steps. Subsequently, phosphonate **6** was reacted with sodium methoxide in anhydrous DMF and a THF solution of acetaldehyde was added. After stirring at room temperature, the mixture was quenched and treated. The crude product was purified by silica gel column chromatography, providing olefin **7** in 37% overall yield from **3**. Notably, this route requires only one chromatographic purification (the last) over four steps, two of which are carried out in bulk, with no solvents added, thus making the overall procedure simple and cost-effective.

### 2.3. Synthesis of (±)-3-Deoxyradicinin (***2***)

Finally, the formation of the second pyrone ring of (±)-3-deoxyradicinin (**2**) was investigated. Preliminary attempts to directly cyclize methoxy alkene **7** in the presence of either crotonic acid or crotonyl chloride and Lewis acids failed, probably because of the low reactivity of the methoxy substituent. Therefore, the methyl protecting group at C-4 was first removed. Several reported *O*-demethylation methods for compound **7** were evaluated; however, only the thermally induced pyrolytic protocol [40], consisting of heating **7** at 70–80 °C for 3 h in the presence of aluminum trichloride and sodium iodide under solvent-free conditions, afforded the demethylated product **8**, as revealed by comparison of ^1^H-NMR of crude with spectrum of pure **8** [23]. Compound **8** was not isolated, but subsequently converted as crude into (±)-3-deoxyradicinin (**2**) by treatment with crotonic acid and phosphorus oxychloride in the presence of ZnCl_2_ under solvent-free conditions (Scheme 1). Compound **2** was thus obtained in 54% yield over the two steps and with >97% purity, as determined by ^1^H-NMR spectroscopy (Figure S10) and GC–MS analysis (Figure S12). Notably, as determined by ^1^H-NMR and GC-MS analyses, the double bond configuration in the final compound **2** is exclusively *E*, indicating that under the reaction conditions also the minor *Z* isomer of **8** undergoes double bond isomerization to the *E* configuration [25]. The procedure described herein then provides (±)-3-deoxyradicinin (**2**) from α-pyrone **3** in six steps and 23% overall yield, thus being significantly more efficient than previously reported syntheses (vide infra).

### 2.4. Comparison with Literature Syntheses of (±)-3-Deoxyradicinin (***2***)

To evaluate the efficacy of the newly developed protocol for the synthesis of **2** a comparison with the previously reported literature procedures is described in Table 2. The first synthetic approach to **2** was reported in 1969 by Yamamura and coworkers [26]. This synthesis affords **2** in 10% overall yield after five steps starting from a not commercially available precursor. The major drawbacks are the use of toxic carbon disulfide and carcinogenic tetrachloroethane as solvents and the use of difficult to handle TiCl_4_, thus hampering its scalability. In a second approach described in 2012 by Nakajima and coworkers [24] the synthesis of **2** was achieved in four steps from **4** in a few mgs scale and the yields were not reported. Moreover, this method employs the air-sensitive NaHMDS and corrosive TiCl_4_ and involves out one step at high temperature in a sealed tube, conditions which hamper its application on a large scale. In 2019 our research group described a more straightforward approach which led to **2** in five steps and a 16% yield [23]. The major drawbacks of this synthesis were again the use of air sensitive TiCl_4_, TMSCl, and LDA as well as the need of very low temperatures in two steps, thus limiting its large-scale use. This synthetic procedure was further optimized by Lockner and coworkers [25], who reduced the number of steps, but at the expense of the overall yield. All these procedures, including the one reported in the present study, afforded less than one gram of the final product **2**. However, the present procedure enables the gram-scale preparation of **7**, the immediate precursor of **2**.

Comparison of these former synthetic procedures with the one reported in this paper highlights that the latter provides a higher overall yield, avoids low temperatures and, in most steps, the use of additional solvents and chromatographic purifications, making the overall procedure potentially scalable to the gram scale. Only the bromination step remains to be improved because, despite many attempts to change the solvent and/or brominating agent, the best results were still obtained with the use of toxic CCl_4_.

## 3. Conclusions

In conclusion, a practical synthetic route to (±)-3-deoxyradicinin (**2**) was developed starting from the inexpensive and commercially available α-pyrone **3**. As an additional outcome of this study, the structurally interesting natural product 5,6-dehydrokawain (**9**) was also obtained. Compound **2**, a biosynthetic precursor of the naturally occurring phytotoxin radicinin (**1**), was selected as the synthetic target because it exhibits biological activity comparable to that of **1** while being considerably easier to prepare. Therefore, **2** may represent a more accessible alternative to naturally occurring radicinin (**1**) for potential applications in both agriculture and medicine.

The overall synthetic strategy was designed according to principles of operational safety and simplicity, with the aim of minimizing the use of hazardous reagents and solvents and avoiding laborious purification procedures. Notably, four of the six synthetic steps were successfully performed on a gram scale, demonstrating the practical applicability of the methodology and its potential for further scale-up. Particular attention was devoted to developing a more environmentally sustainable protocol for the allylic bromination of **4**. However, none of the greener conditions investigated afforded satisfactory results, making it necessary to employ a Wohl–Ziegler radical bromination mediated by NBS. This reaction provided bromopyrone **5** with satisfactory regioselectivity; nevertheless, its reliance on toxic CCl_4_ remains a major limitation to the environmental sustainability of the overall synthetic procedure.

The successful preparation of (±)-3-deoxyradicinin (**2**) in 23% overall yield establishes the feasibility of the proposed synthetic strategy. Further improvements could be achieved by developing a greener bromination protocol and a practical procedure for scaling up the final two steps involved in constructing the second 4-pyranone ring. Overall, this work provides a practical route to **2** and lays the groundwork for the future preparation of structural analogues and for larger-scale production aimed at biological and agrochemical investigations. The increased accessibility of **2** could also facilitate further comparative studies of its biological activity and that of naturally occurring radicinin (**1**), as well as more detailed investigations into its mechanism of action [41].

## 4. Materials and Methods

### 4.1. General Experimental Procedures

Melting points were determined with a Kofler hot-stage apparatus and are reported uncorrected. ^1^H (400 MHz) and ^13^C (100 MHz) NMR spectra were recorded using a Varian (Palo Alto, CA, USA) INOVA 400 spectrometer. GC-MS spectra were obtained using a Hewlett-Packard (Palo Alto, CA, USA) HP 6890 gas chromatograph equipped with an HP-5975 mass spectrometric detector. HRESI mass spectra were obtained by direct injection into an Agilent Technologies (Santa Clara, CA, USA) 1100 LC/MS TOF instrument in positive mode. Analytical TLC was carried out on Macherey–Nagel (Dueren, Germany) silica gel 60 sheets. The spots were visualized by exposing the plates to UV radiation (254 nm) and/or by spraying them with a potassium permanganate solution. Column chromatography was performed using Merck (Darmstadt, Germany) silica gel Kieselgel 60, 60–230 mesh. All analytical-grade solvents and reagents, including starting compound **3** (98% purity), were purchased from Sigma-Aldrich (St. Louis, MO, USA) and used without further purification. NBS was recrystallized from water and dried before use. THF was freshly distilled before use over sodium benzophenone ketyl under a nitrogen atmosphere. The completion of the reactions was assessed by monitoring the reaction mixture by TLC and, when possible, by GC–MS analysis. The *E:Z* ratio of compound **7** was determined by comparing the integrals of the ^1^H-NMR vinyl signals of this compound at 5.97 ppm (*E* isomer) and at 5.89 ppm (*Z* isomer) and confirmed by GC-MS. The conversions and the product ratios reported in Table S2 were first determined by monitoring the reaction by GC-MS analysis and then confirmed by 1H NMR signals integration on the crude. The reported isolated yields represent single experiments. The radical bromination was performed in a well-ventilated fume hood with appropriate PPE due to the hazards associated with CCl_4_ and NBS. Moisture-sensitive AlCl_3_ and POCl_3_ were handled with appropriate PPE under a well-ventilated fume hood.

### 4.2. 4-Methoxy-6-methyl-2H-pyran-2-one (***4***) [42]

To a solution of 4-hydroxy-6-methyl-2*H*-pyran-2-one **3** (1.00 g, 7.93 mmol) in trimethyl phosphate (3.8 mL), potassium carbonate (1.3 g) was added. The reaction mixture was heated to reflux under vigorous stirring for one hour. Upon complete consumption of the starting material the reaction was quenched by the addition of water (20 mL). The resulting precipitate was filtered and recrystallized from ethanol, affording 435 mg of pure compound **4** as white crystals. The mother liquors were concentrated again under reduced pressure, obtaining a white precipitated which was collected by vacuum filtration and washed with a small amount of cold ethanol, providing an additional 560 mg of purified product. In total, 995 mg (7.10 mmol, 90% yield) of compound **4** were isolated. M.p. 85–88 °C (lit. [43] m.p. 89 °C). ^1^H-NMR (400 MHz, CDCl_3_) δ (ppm) 2.21 (s, 3H), 3.80 (s, 3H), 5.42 (d, *J* = 1.6 Hz, 1H), 5.79 (d, *J* = 1.6 Hz, 1H). ^13^C-NMR (100 MHz, CDCl_3_) δ (ppm) 20.0, 56.0, 87.5, 100.5, 162.2, 165.2, 171.5. MS (EI) *m*/*z* 140 (M^+^, 54), 125 (52), 112 (100), 69 (73), 43 (36). HRESIMS (+) *m*/*z* 141.0555 [M + H]^+^ (calcd for C_7_H_9_O_3_ 141.0552).

### 4.3. 6-(Bromomethyl)-4-methoxy-2H-pyran-2-one (***5***) [44]

To a solution of compound **4** (330 mg, 2.36 mmol) in carbon tetrachloride (59 mL), NBS (460 mg, 2.6 mmol) was added. The reaction mixture was heated to reflux, irradiated with a visible-light lamp (60 W), and stirred under these conditions for one hour. The reaction was then stopped by removing the solvent under reduced pressure. The crude product was purified by silica gel column chromatography (*n*-hexane/ethyl acetate 6:4), affording 340 mg (1.55 mmol, 66% yield) of bromopyrone **5** as a dark liquid. ^1^H-NMR (400 MHz, CDCl_3_) δ (ppm) 3.83 (s, 3H), 4.13 (s, 2H), 5.51 (s, 1H), 6.11 (s, 1H). ^13^C-NMR (100 MHz, CDCl_3_) δ (ppm) 26.7, 56.3, 89.6, 102.4, 158.6, 163.6, 170.5. MS (EI) *m*/*z* 220 (M^+^, 15), 218 (M^+^, 15), 125 (76), 111 (100), 69 (37), 59 (21). HRESIMS (+) *m*/*z* 218.9654 [M + H]^+^ (calcd for C_7_H_8_BrO_3_ 218.9657).

### 4.4. Dimethyl ((4-Methoxy-2-oxo-2H-pyran-6-yl)methyl)phosphonate (***6***) [44]

Bromopyrone **5** (340 mg, 1.55 mmol) was dissolved in trimethyl phosphite (320 μL). The reaction mixture was heated at 60 °C for 5 h. Upon completion of the reaction the crude product was purified by silica gel column chromatography using a gradient of elution: ethyl acetate/*n*-hexane 2:1, then ethyl acetate and finally ethyl acetate/methanol 96:4. This procedure afforded 358 mg (1.44 mmol, 93% yield) of compound **6** as a deliquescent solid. ^1^H-NMR (400 MHz, CDCl_3_) δ (ppm) 3.06 (d, ^2^*J*_H-P_ = 21.6 Hz, 2H), 3.80 (s, 3H), 3.81 (s, 3H), 3.83 (s, 3H), 5.46 (s, 1H), 6.02 (s, 1H). ^13^C-NMR (100 MHz, CDCl_3_) δ (ppm) 31.3 (d, ^1^*J*_C-P_ = 140.3 Hz), 53.3 (d, ^2^*J*_C-P_ = 6.7 Hz), 56.0, 88.3, 102.7, 155.7 (d, ^2^*J*_C-P_ = 9.8 Hz), 164.0, 170.8. MS (EI) *m*/*z* 248 (M^+^, 39), 220 (100), 125 (63), 111 (62), 109 (24), 69 (28), 59 (13). HRESIMS (+) *m*/*z* 249.0528 [M + H]^+^ (calcd for C_9_H_14_O_6_P 249.0533).

### 4.5. (E)-4-Methoxy-6-(prop-1-en-1-yl)-2H-pyran-2-one (***7***) [24]

Sodium methoxide (17 mg) was dissolved in anhydrous DMF (700 μL) under a nitrogen atmosphere, then substrate **6** (72 mg, 0.29 mmol) was added. After five minutes, 400 μL of a 1 M solution of acetaldehyde in anhydrous THF were added and the reaction mixture was stirred at room temperature for 30 min. Upon completion, the reaction was quenched with cold water (2 mL) and extracted three times with ethyl acetate. The combined organic layers were washed with brine, dried over anhydrous sodium sulfate, and concentrated under reduced pressure. The crude product was purified by silica gel column chromatography (*n*-hexane/ethyl acetate 1:1), affording product **7** (36.6 mg, 0.22 mmol, 76% yield) as a yellow solid with a 96:4 *E:Z* ratio. M.p. 72–75 °C. *E* isomer: ^1^H-NMR (400 MHz, CDC_l3_) δ (ppm) 1.90 (d, *J* = 6.8 Hz, 3H), 3.80 (s, 3H), 5.44 (s, 1H), 5.75 (s, 1H), 5.97 (d, *J* = 15.6 Hz, 1H), 6.71 (m, 1H). ^13^C-NMR (100 MHz, CDCl_3_) δ (ppm) 18.7, 57.4, 89.5, 93.8, 122.5, 137.3, 159.1, 160.2, 167.1. MS (EI) *m*/*z* 166 (M^+^, 61), 138 (100), 125 (18), 109 (15), 95 (31), 69 (56). *Z* isomer: ^1^H-NMR (400 MHz, CDCl_3_) δ (ppm) 2.13 (d, *J* = 7.2 Hz, 3H), 3.81 (s, 3H), 5.46 (s, 1H), 5.86 (s, 1H), 5.89 (d, *J* = 12.0 Hz, 1H), 6.02 (m, 1H). MS (EI) *m*/*z* 166 (M^+^, 82), 138 (100), 125 (20), 109 (20), 95 (31), 69 (72). HRESIMS (+) *m*/*z* 167.0708 [M + H]^+^ (calcd for C_9_H_11_O_3_ 167.0704).

### 4.6. Large-Scale Synthesis of Pyrone (***7***)

A solution of compound **3** (4.0 g, 31.7 mmol) in trimethyl phosphate (7.4 mL) was treated with potassium carbonate (5.2 g). The reaction mixture was heated at reflux for one hour. Upon completion, the reaction was quenched with cold water (10 mL) to dissolve the remaining carbonate. The resulting precipitate was collected by vacuum filtration and washed with cold ethanol (5 mL). This procedure afforded 4.0 g (28.5 mmol, 90% yield) of compound **4**. A solution of **4** (4 g, 28.5 mmol) in carbon tetrachloride (640 mL) was treated with NBS (5.20 g, 29.2 mmol) and dibenzoyl peroxide (692 mg, 2.9 mmol) as a radical initiator. The reaction mixture was heated at reflux for 3 h. The reaction was then quenched with cold water (160 mL). After phase separation, the aqueous layer was extracted three times with dichloromethane. The combined organic phases were dried over anhydrous sodium sulfate, filtered, and concentrated under reduced pressure, providing bromopyrone **5** (3.96 g, 18.1 mmol). Crude **5** was dissolved in trimethyl phosphite (5 mL) and the reaction mixture was heated at 60 °C for 5 h. Upon completion of the reaction, the crude product was filtered through a silica plug by eluting with ethyl acetate:*n*-hexane 2:1, then with ethyl acetate and finally with ethyl acetate:methanol 96:4, affording compound **6** (4.20 g, 16.9 mmol) in an overall 60% yield over the last two steps. To a stirred solution of sodium methoxide (1.0 g, 18.5 mmol) in anhydrous DMF (45 mL), mantained under nitrogen atmosphere, substrate **6** was added. To this mixture, 12.5 mL of a 2 M solution of acetaldehyde in THF was added. The reaction was stirred at room temperature for 30 min. Upon completion, the mixture was concentrated under reduced pressure, diluted with cold water (40 mL), and extracted three times with ethyl acetate. The organic layer was washed with brine, dried over anhydrous sodium sulfate and the solvent was evaporated under reduced pressure. The crude product was purified by silica gel column chromatography, eluting first with *n*-hexane/ethyl acetate (1:1) and then with pure ethyl acetate, affording olefin **7** (1.94 g, 11.7 mmol) in 69% yield with a 96:4 *E*:*Z* ratio. Olefin **7** was obtained in 37% overall yield from **3**.

### 4.7. (±)-3-Deoxyradicinin (***2***) [23]

Alkene **7** (450 mg, 2.71 mmol), AlCl_3_ (720 mg, 2 eq.) and NaI (812 mg, 2 eq.) were mixed for a few minutes under vigorous stirring. The resulting solid mixture was heated at 70–80 °C for 3 h. Afterwards, the mixture was cooled and treated with a 5% aqueous solution of sodium metabisulfite (7.5 mL). The aqueous layer was extracted three times with chloroform. The organic layer was dried over anhydrous sodium sulfate, filtered under vacuum, and the solvent was removed under reduced pressure. The resulting demethylated olefin **8** was used directly in the subsequent reaction without any chromatographic purification. Crude compound **8** was treated with phosphorus oxychloride (POCl_3_, 2.00 mL) and zinc chloride (ZnCl_2_, 1.45 g). Subsequently, crotonic acid (242 mg, 2.8 mmol) was added. The reaction mixture was refluxed for approximately 4 h and, after cooling, was quenched with a 1.0 M aqueous solution of sodium carbonate (70 mL) and ice. The aqueous layer was extracted three times with dichloromethane. The organic layer was dried over anhydrous sodium sulfate, filtered under vacuum, and the solvent removed under reduced pressure, yielding 800 mg of crude product, which was purified by silica gel column chromatography, eluting with dichloromethane and then with dichloromethane:MeOH 10:1. Racemic deoxyradicinin (**2**) was thus obtained (319 mg, 1.45 mmol, 54% yield over two steps). The purity of isolated **2** was estimated to be >97% based on its ^1^H-NMR spectrum (Figure S10) and GC–MS chromatogram (Figure S12). M.p. 143–145 °C. ^1^H-NMR (400 MHz, CDCl_3_) δ (ppm) 1.54 (d, *J* = 6.4 Hz, 3H), 1.95 (d, *J* = 6.8 Hz, 3H), 2.61 (m, 2H), 4.75 (m, 1H), 5.83 (s, 1H), 6.02 (d, *J* = 15.6 Hz, 1H), 6.92 (m, 1H). ^13^C-NMR (100 MHz, CDCl_3_) δ (ppm) 18.8, 20.4, 43.9, 76.7, 98.3, 100.3, 122.8, 140.2, 157.4, 163.6, 176.1, 186.6. MS (EI) *m*/*z* 220 (M^+^, 42), 205 (100), 179 (21), 69 (79), 41 (28). HRESIMS (+) *m*/*z* 221.0811 [M + H]^+^ (calcd for C_12_H_13_O_4_ 221.0814).

### 4.8. 5,6-Dehydrokawain (***9***) [30,31]

Methyl ether **4**, (50 mg, 0.36 mmol) was dissolved in 900 µL of anhydrous DMF. Subsequently, sodium *t*-butoxide (38 mg, 1.1 eq) and benzaldehyde (40.2 µL, 1.1 eq) were added to the solution. The latter was stirred at room temperature for 30 min. Upon completion, the reaction was quenched by the addition of 3.5 mL of cold water. The obtained precipitate was filtered and washed, in turn, with cold water and petroleum ether providing compound **9** (47 mg, 0.21 mmol, 57% yield). M.p. 138–139 °C (lit. [30] m.p. 137–138 °C). ^1^H-NMR (400 MHz, CDCl_3_) δ (ppm) 3.82 (s, 3H), 5.49 (d, J = 2.0 Hz, 1H), 5.94 (d, J = 2.4 Hz,1H), 6.58 (d, J = 16.0 Hz, 1H), 7.36 (m,3H), 7.50 (m,3H). ^13^C-NMR (100 MHz, CDCl_3_) δ (ppm) 56.0, 88.9, 101.4, 118.6, 127.4, 128.9,129.5, 135.2, 135.8, 158.6, 164.0, 171.1. MS (EI) *m*/*z* 228 (M^+^,100), 200 (32), 185 (16), 157 (47), 129 (22), 103 (18), 77 (19). HRESIMS (+) *m*/*z* 229.0861 [M + H]^+^ (calcd for C_14_H_13_O_3_ 229.0865).

## Figures and Tables

**Figure 1:**
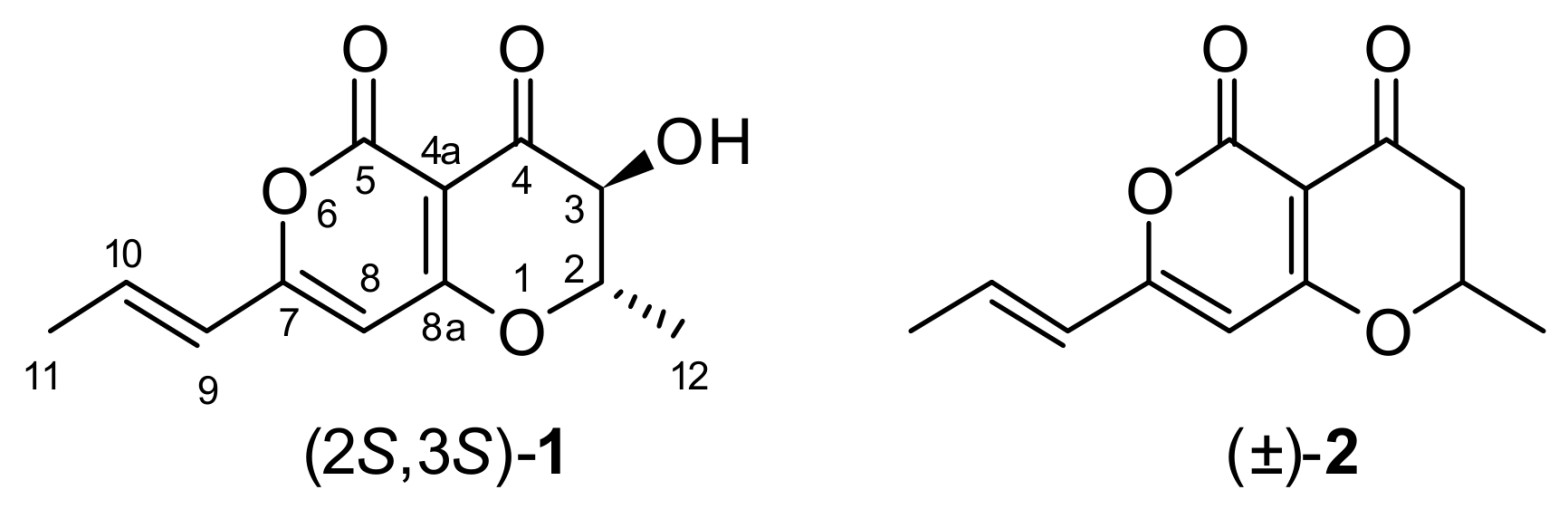
Structures of radicinin (**1**) and (±)-3-deoxyradicinin (**2**).

**Scheme 1:**
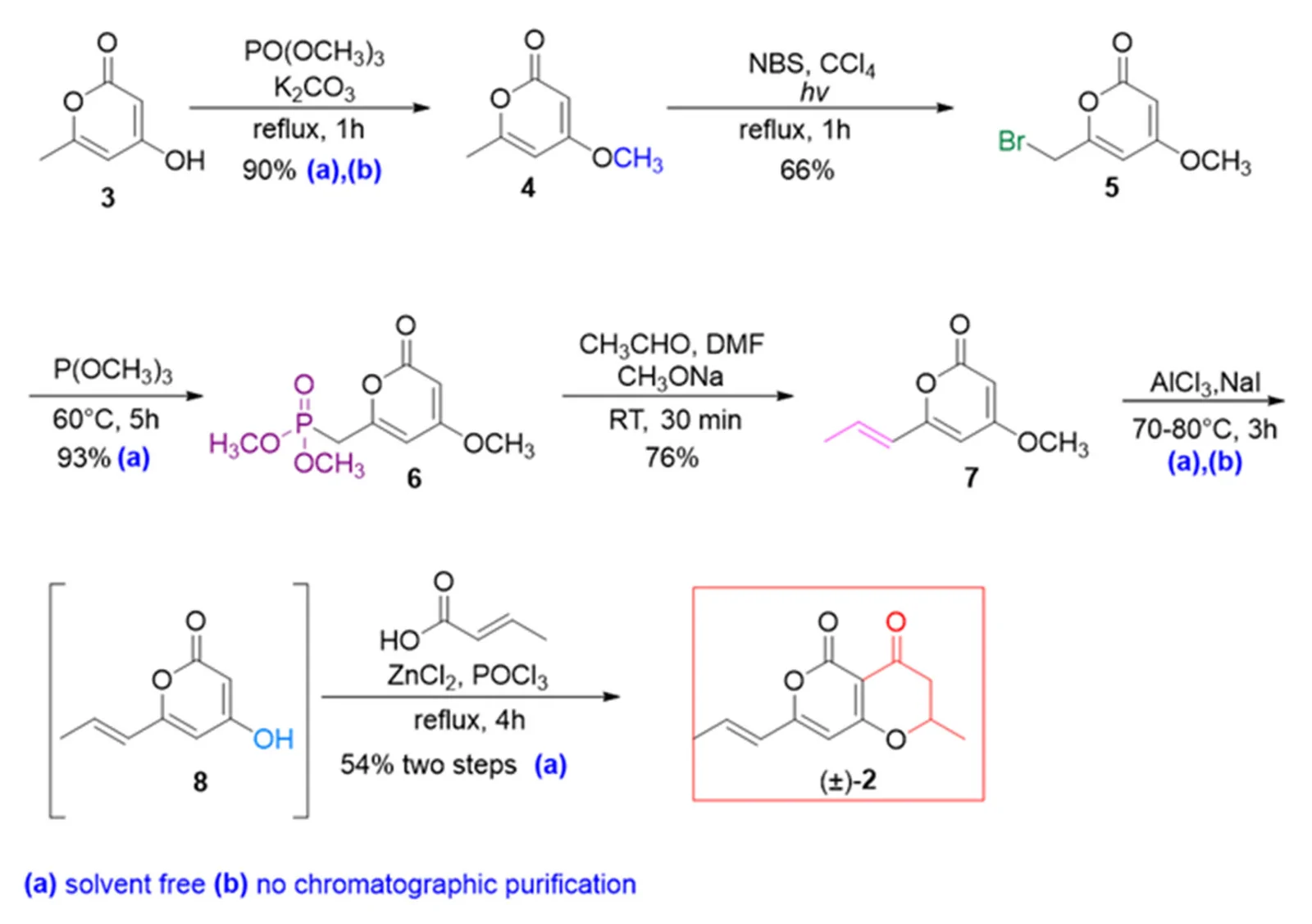
General overview of the small-scale synthetic methodology for preparation of (±)-3-deoxyradicinin (**2**).

**Scheme 2:**
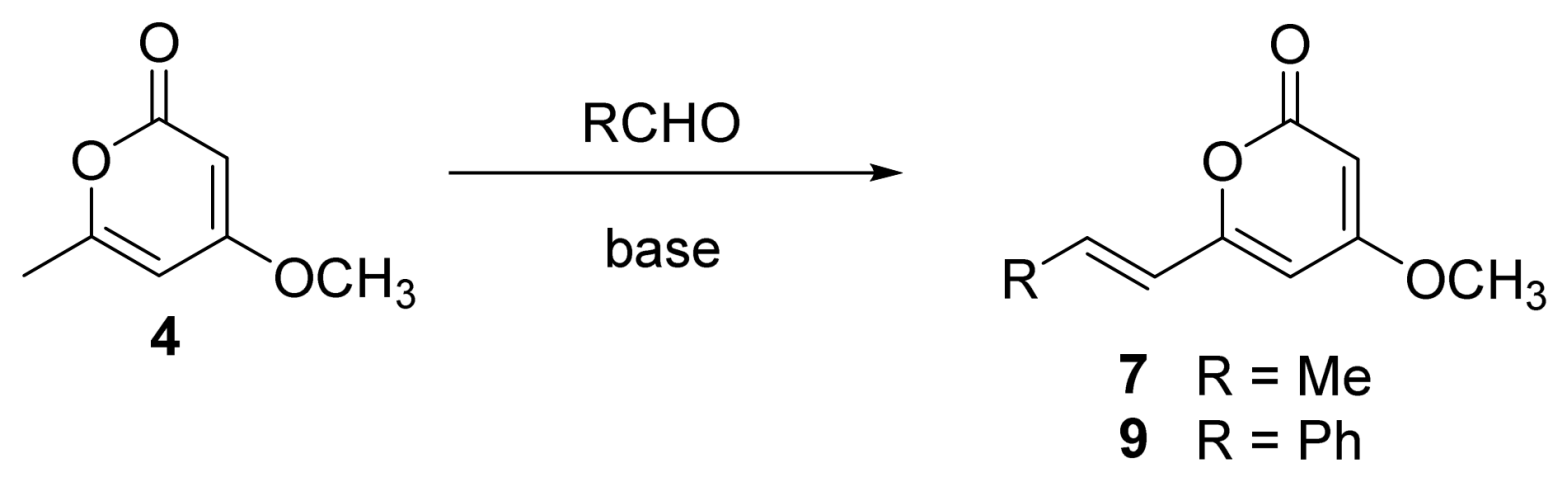
Synthesis of 6-alkenyl pyran-2-ones by 6-methyl deprotonation and condensation with aldehydes.

**Scheme 3:**
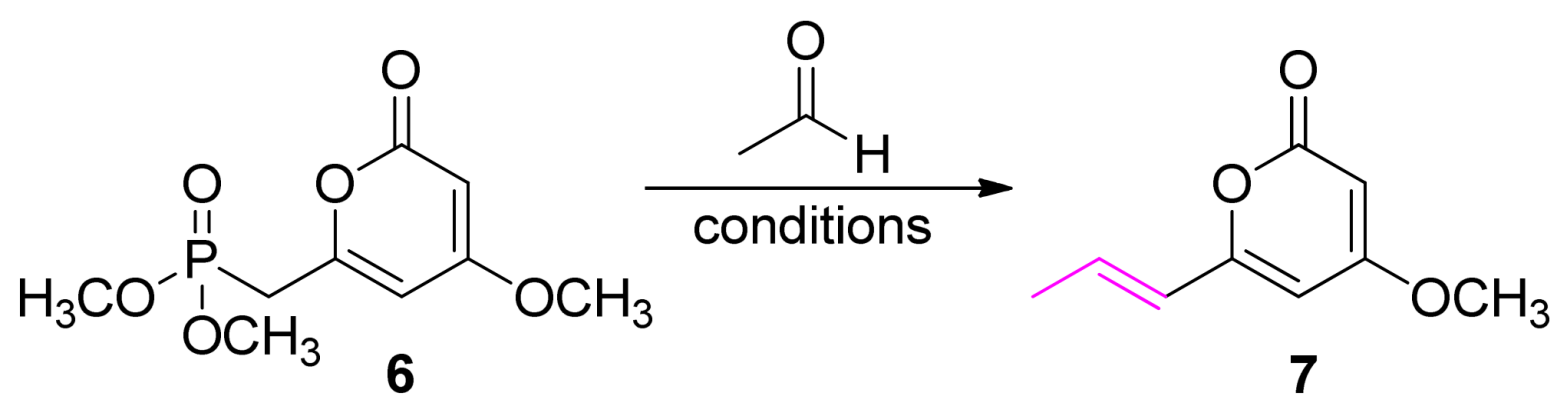
HWE reaction on phosphonate **6**.

**Table 1:** Experimental conditions and results for the synthesis of **7** via the HWE reaction.

Entry	Base	Solvent	Temperature (°C)	Time (h)	Yield (%) ^a^	*E*:*Z* Ratio ^b^
1	NaH	THF	0	1	86	77:23
2	MeONa	DMF	0	1	86	77:23
3	NaHMDS	THF	RT	1	36	80:20
4	NaH	THF	RT	0.5	93	83:17
5	MeONa	DMF	RT	0.5	76	96:4
6	NaH	THF	RT	1	80	80:20

^a^ Isolated yield after column chromatography. ^b^ Determined by ^1^H-NMR and GC-MS analyses.

**Table 2:** Comparison of yields and operation conditions with literature procedures.

Starting Material	N. Steps	Overall Yield (%)	Amount of 2 Obtained	Toxic or Air Sensitive Reagents or Solvents	Comments	Ref.
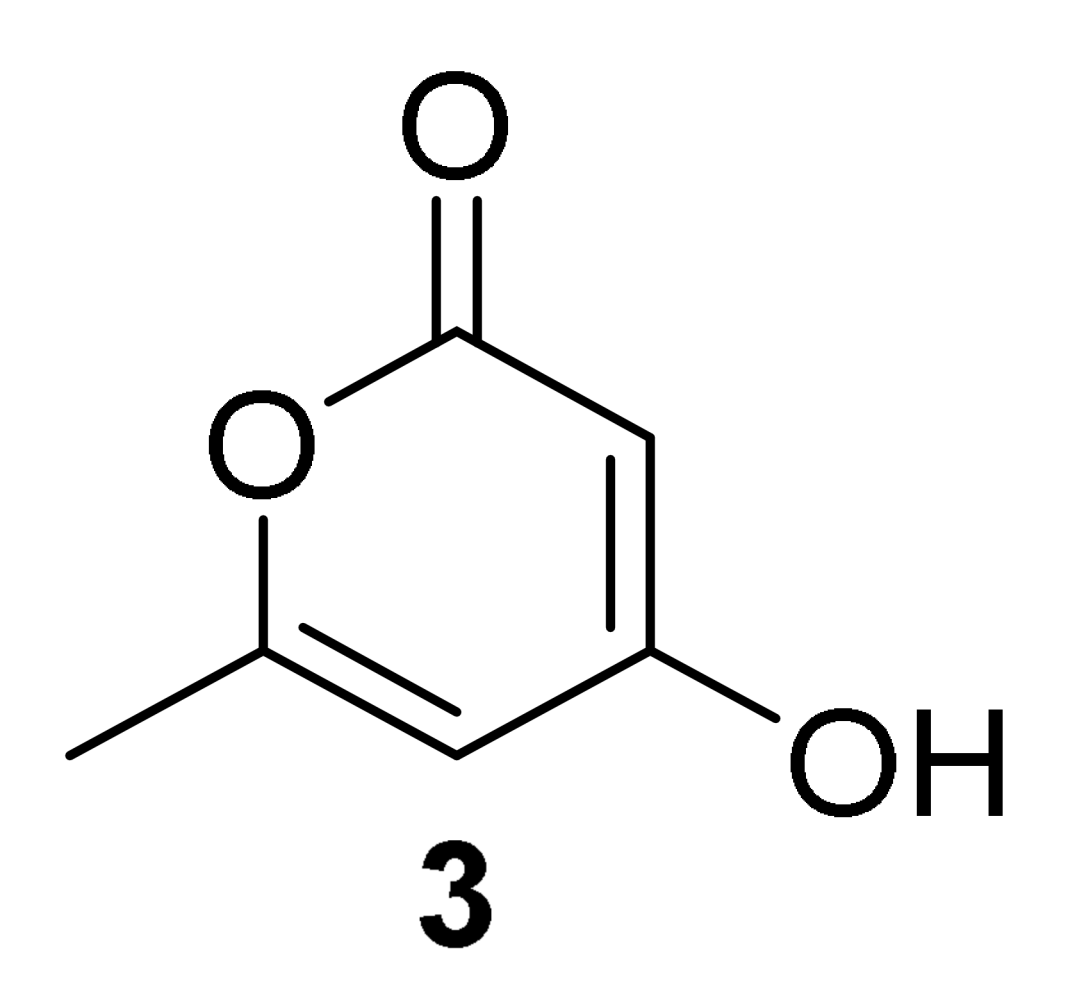	6	23	319 mg (1.94 g for intermediate **7**)	CCl_4_, AlCl_3_, POCl_3_	No low temperatures, some steps in bulk and without chromatography	this study
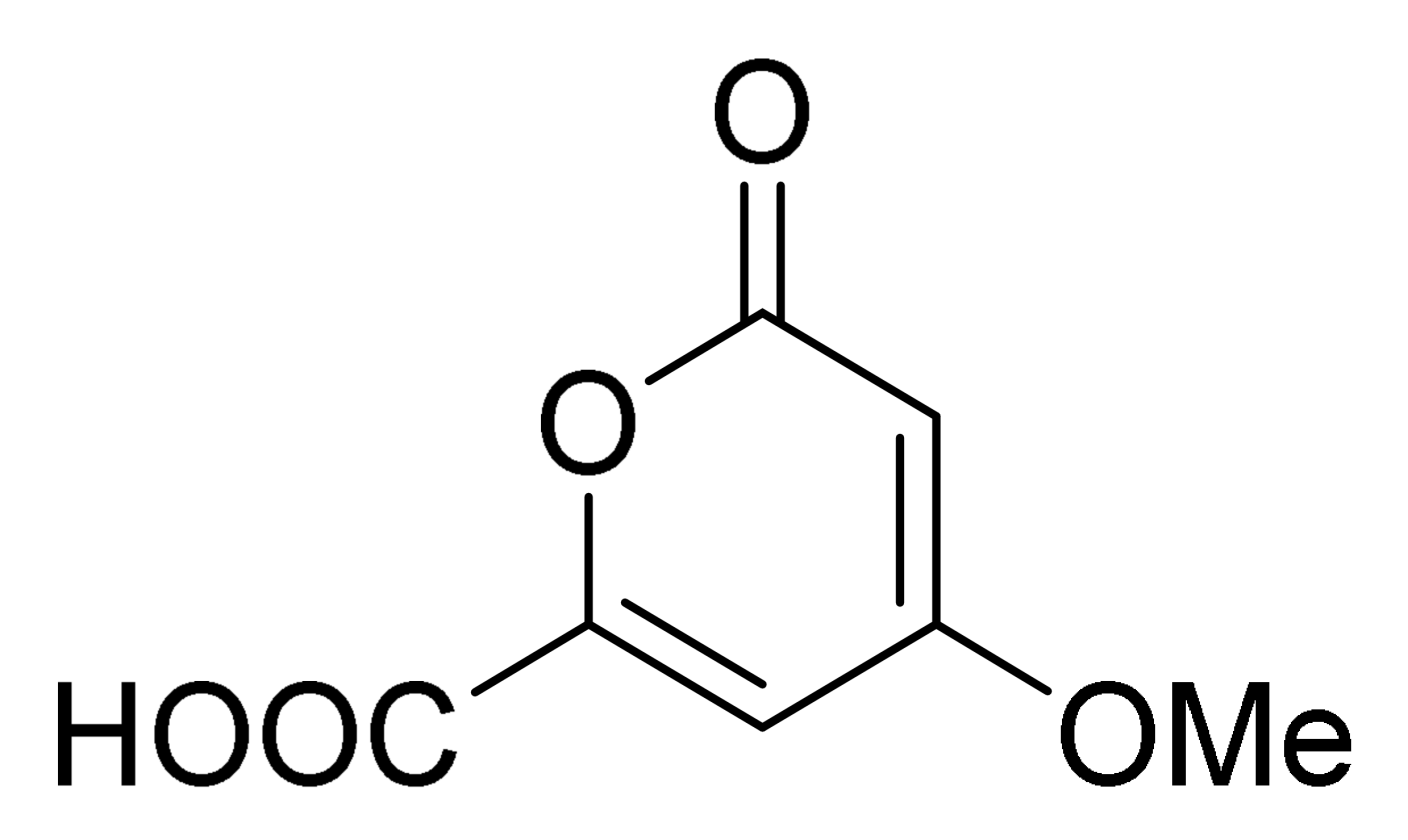	5	10	500 mg	CS_2_, TiCl_4_ CHCl_2_CHCl_2_	Not commercially available starting reagent	[26]
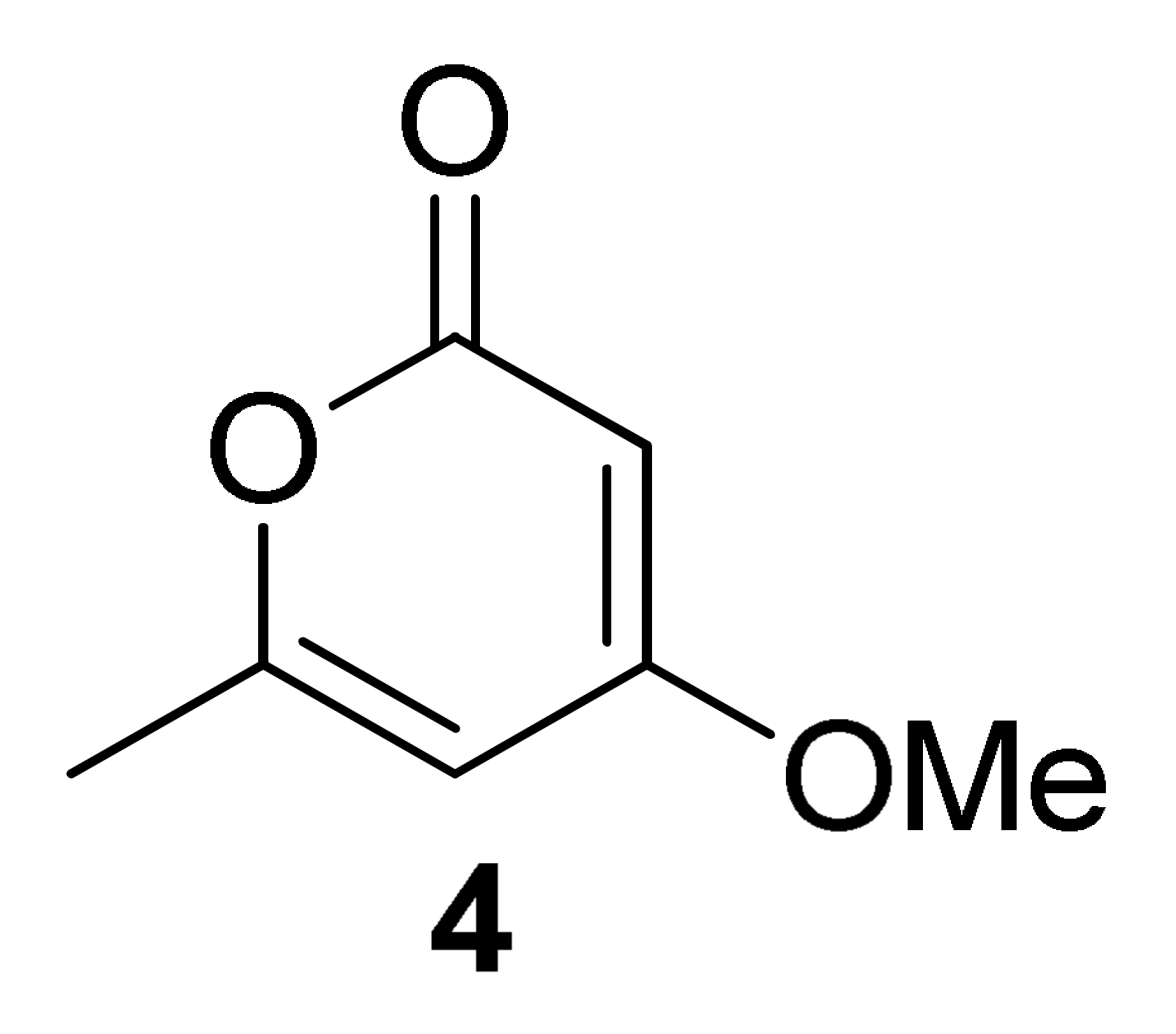	4	Not reported	<10 mg	NaHMDS, TiCl_4_, CH_2_Cl_2_	Reaction in sealed tube at 160 °C	[24]
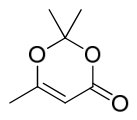	5	16	130 mg	LDA, TMSCl, TiCl_4_, CH_2_Cl_2_	Low temperature (−78 °C) in two steps	[23]
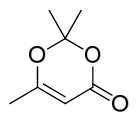	3	7.5	91 mg	LiHMDS, Et_2_Zn, POCl_3_	Low temperature (−78 °C) in one step	[25]

## Data Availability

The original contributions presented in this study are included in the article/Supplementary Materials. Further inquiries can be directed to the corresponding authors.

## Supplementary Materials

The following supporting information can be downloaded at: https://www.mdpi.com/article/10.3390/toxins18100412/s1, Table S1: Conditions for screening the direct side-chain elongation of compound **4**; Table S2: Conditions for screening the benzylic bromination of **4**; Figure S1: ^1^H-NMR spectrum (400 MHz, CDCl_3_) of 4-methoxy-6-methyl-2*H*-pyran-2-one (**4**)*;* Figure S2: ^13^C-NMR spectrum (100 MHz, CDCl_3_) of 4-methoxy-6-methyl-2*H*-pyran-2-one (**4**); Figure S3: ^1^H-NMR spectrum (400 MHz, CDCl_3_) of 6-(bromomethyl)-4-methoxy-2*H*-pyran-2-one (**5**); Figure S4: ^13^C-NMR spectrum (100 MHz, CDCl_3_) of 6-(bromomethyl)-4-methoxy-2*H*-pyran-2-one (**5**); Figure S5: ^1^H-NMR spectrum (400 MHz, CDCl_3_) of dimethyl ((4-methoxy-2-oxo-2*H*-pyran-6-yl)methyl)phosphonate (**6**); Figure S6: ^13^C-NMR spectrum (100 MHz, CDCl_3_) of dimethyl ((4-methoxy-2-oxo-2*H*-pyran-6-yl)methyl)phosphonate (**6**); Figure S7: ^1^H-NMR spectrum (400 MHz, CDCl_3_) of (*E*)-4-methoxy-6-(prop-1-en-1-yl)-2*H*-pyran-2-one (**7**); Figure S8: ^13^C-NMR spectrum (100 MHz, CDCl_3_) of (*E*)-4-methoxy-6-(prop-1-en-1-yl)-2*H*-pyran-2-one (**7**); Figure S9: GC-MS chromatogram of olefin **7**. Peak 1 corresponds to the *Z* isomer, while peak 2 corresponds to the *E* isomer. The *E:Z* ratio was determined to be 96:4; Figure S10: ^1^H-NMR spectrum (400 MHz, CDCl_3_) of (±)-3-deoxyradicinin (**2**); Figure S11: ^13^C-NMR spectrum (100 MHz, CDCl_3_) of (±)-3-deoxyradicinin (**2**); Figure S12: GC-MS chromatogram of (±)-3-deoxyradicinin (**2**); Figure S13: ^1^H-NMR spectrum (400 MHz, CDCl_3_) of 5,6-dehydrokawain (**9**); Figure S14: ^13^C-NMR spectrum (100 MHz, CDCl_3_) of 5,6-dehydrokawain (**9**).

## References

[1] Walton J.D., Panaccione D.G. (1993). Host-Selective Toxins and Disease Specificity: Perspectives and Progress. Annu. Rev. Phytopathol..

[2] Wolpert T.J., Dunkle L.D., Ciuffetti L.M. (2002). Host-Selective Toxins and Avirulence Determinants: What’s in a Name?. Annu. Rev. Phytopathol..

[3] Dayan F.E., Duke S.O. (2014). Natural Compounds as Next-Generation Herbicides. Plant Physiol..

[4] Gerwick B.C., Sparks T.C. (2014). Natural Products for Pest Control: An Analysis of Their Role, Value and Future. Pest Manag. Sci..

[5] Cimmino A., Masi M., Evidente M., Superchi S., Evidente A. (2015). Fungal Phytotoxins with Potential Herbicidal Activity: Chemical and Biological Characterization. Nat. Prod. Rep..

[6] Evidente A., Kornienko A., Cimmino A., Andolfi A., Lefranc F., Mathieu V., Kiss R. (2014). Fungal Metabolites with Anticancer Activity. Nat. Prod. Rep..

[7] Butler M.S., La Clair J.J. (2026). The Role of Natural Product Chemistry in Drug Discovery: Two Decades of Progress and Perspectives. J. Nat. Prod..

[8] Gakuubi M.M., Ching K.C., Munusamy M., Wibowo M., Liang Z.-X., Kanagasundaram Y., Ng S.B. (2022). Enhancing the Discovery of Bioactive Secondary Metabolites From Fungal Endophytes Using Chemical Elicitation and Variation of Fermentation Media. Front. Microbiol..

[9] Clarke D.D., Nord F.F. (1953). Radicinin: A New Pigment from *Stemphylium radicinum*. Arch. Biochem. Biophys..

[10] Robeson D.J., Gray G.R., Strobel G.A. (1982). Production of the Phytotoxins Radicinin and Radicinol by *Alternaria chrysanthemi*. Phytochemistry.

[11] Nukina M., Marumo S. (1977). Radicinol, a New Metabolite of *Cochliobolus lunatus*, and Absolute Stereochemistry of Radicinin. Tetrahedron Lett..

[12] Tal B., Robeson D.J., Burke B.A., Aasen A.J. (1985). Phytotoxins from *Alternaria helianthi*: Radicinin, and the Structures of Deoxyradicinol and Radianthin. Phytochemistry.

[13] Noordeloos M.E., De Gruyter J., Van Eijk G.W., Roeijmans H.J. (1993). Production of Dendritic Crystals in Pure Cultures of *Phoma* and *Ascochyta* and Its Value as a Taxonomic Character Relative to Morphology, Pathology and Cultural Characteristics. Mycol. Res..

[14] Kadam S., Poddig J., Humphrey P., Karwowski J., Jackson M., Tennent S., Fung L., Hochlowski J., Rasmussen R., McAlpine J. (1994). Citrinin Hydrate and Radicinin: Human Rhinovirus 3C-Protease Inhibitors Discovered in a Target-Directed Microbial Screen. J. Antib..

[15] Nakajima H., Ishida T., Otsuka Y., Hamasaki T., Ichinoe M. (1997). Phytotoxins and Related Metabolites Produced by *Bipolaris coicis*, the Pathogen of Job’s Tears. Phytochemistry.

[16] Pryor B.M., Gilbertson R.L. (2002). Relationships and Taxonomic Status of *Alternaria radicina*, *A. carotiincultae*, and *A. petroselini* Based upon Morphological, Biochemical, and Molecular Characteristics. Mycologia.

[17] Grove J.F. (1964). Metabolic Products of *Stemphylium radicinum*. Part I. Radicinin. J. Chem. Soc..

[18] Masi M., Freda F., Sangermano F., Calabrò V., Cimmino A., Cristofaro M., Meyer S., Evidente A. (2019). Radicinin, a Fungal Phytotoxin as a Target-Specific Bioherbicide for Invasive buffelgrass (*Cenchrus ciliaris*) Control. Molecules.

[19] Li S., Shao M.-W., Lu Y.-H., Kong L.-C., Jiang D.-H., Zhang Y.-L. (2014). Phytotoxic and Antibacterial Metabolites from *Fusarium proliferatum* ZS07 Isolated from the Gut of Long-Horned Grasshoppers. J. Agric. Food Chem..

[20] Hussain H., Kliche-Spory C., Al-Harrasi A., Al-Rawahi A., Abbas G., Green I.R., Schulz B., Krohn K., Shah A. (2014). Antimicrobial Constituents from Three Endophytic Fungi. Asian Pac. J. Trop. Med..

[21] Aldrich T.J., Rolshausen P.E., Roper M.C., Reader J.M., Steinhaus M.J., Rapicavoli J., Vosburg D.A., Maloney K.N. (2015). Radicinin from *Cochliobolus* sp. Inhibits *Xylella fastidiosa*, the Causal Agent of Pierce’s Disease of Grapevine. Phytochemistry.

[22] Mathieu V., Superchi S., Masi M., Scafato P., Kornienko A., Evidente A. (2022). In Vitro Effects of Fungal Phytotoxins on Cancer Cell Viability: First Insight into Structure Activity Relationship of a Potent Metabolite of *Cochliobolus australiensis* Radicinin. Toxins.

[23] Marsico G., Ciccone M.S., Masi M., Freda F., Cristofaro M., Evidente A., Superchi S., Scafato P. (2019). Synthesis and Herbicidal Activity Against buffelgrass (*Cenchrus ciliaris*) of (±)-3-Deoxyradicinin. Molecules.

[24] Suzuki M., Sakuno E., Ishihara A., Tamura J., Nakajima H. (2012). Conversions of Deoxyradicinin to Radicinin and of Radicinin to 3-Epi-Radicinin in the Phytopathogenic Fungus *Bipolaris coicis*. Phytochemistry.

[25] Brandenburg C.A., Castro C.A., Blacutt A.A., Costa E.A., Brinton K.C., Corral D.W., Drozd C.L., Roper M.C., Rolshausen P.E., Maloney K.N. (2020). Synthesis of Deoxyradicinin, an Inhibitor of *Xylella fastidiosa* and *Liberibacter crescens*, a Culturable Surrogate for *Candidatus Liberibacter asiaticus*. J. Nat. Prod..

[26] Kato K., Hirata Y., Yamamura S. (1969). Syntheses of (±)-Radicinin and (±)-Dihydroradicinin. J. Chem. Soc. C.

[27] Clarke S.L., McGlacken G.P. (2015). Access to Trifluoromethylated 4-Alkoxy-2-Pyrones, Pyridones and Quinolones. Tetrahedron.

[28] Saidi M.R., Rajabi F. (2003). A New Protocol for O-Methylation of Phenolic Compounds with Trimethyl Phosphite or Trimethyl Phosphate Under Solvent-Free Condition and Microwave Irradiation. Phosphorus Sulfur Silicon Relat. Elem..

[29] Tang Y., Yu B. (2022). A Mild Heteroatom (*O*-, *N*-, and *S*-) Methylation Protocol Using Trimethyl Phosphate (TMP)-Ca(OH)_2_ Combination. Synthesis.

[30] Kumagai M., Nishikawa K., Mishima T., Yoshida I., Ide M., Koizumi K., Nakamura M., Morimoto Y. (2017). Synthesis of Novel 5,6-Dehydrokawain Analogs as Osteogenic Inducers and Their Action Mechanisms. Bioorg. Med. Chem. Lett..

[31] Kumagai M., Mishima T., Watanabe A., Harada T., Yoshida I., Fujita K., Watai M., Tawata S., Nishikawa K., Morimoto Y. (2016). 5,6-Dehydrokawain from *Alpinia zerumbet* Promotes Osteoblastic MC3T3-E1 Cell Differentiation. Biosci. Biotechnol. Biochem..

[32] Zahra M.H., Salem T.A.R., El-Aarag B., Yosri N., EL-Ghlban S., Zaki K., Marei A.H., Abd El-Wahed A., Saeed A., Khatib A. (2019). *Alpinia zerumbet* (Pers.): Food and Medicinal Plant with Potential In Vitro and In Vivo Anti-Cancer Activities. Molecules.

[33] Obi G., Van Heerden F.R. (2018). Synthesis of 5,6-Dehydrokawain and Some Fluorinated Analogues. Synth. Commun..

[34] Samala R., Basu M.K., Mukkanti K. (2022). Regioselective Functionalization of Pyrones: Facile Synthesis of 6-Styrylpyrones via KHMDS-Mediated Aldol Condensation. Tetrahedron Lett..

[35] De Souza S.P.L., Da Silva J.F.M., De Mattos M.C.S. (2003). A Green Preparation of ***N***-Chloro- and ***N***-Bromosaccharin. Synth. Commun..

[36] Kaur K., Srivastava S. (2020). Artificial Sugar Saccharin and Its Derivatives: Role as a Catalyst. RSC Adv..

[37] De Almeida L.S., Esteves P.M., De Mattos M.C.S. (2015). Tribromoisocyanuric Acid as a Green Reagent for Benzylic Bromination of Alkylarenes. Tetrahedron Lett..

[38] Marcos F.C., Neo A.G., Díaz J., Martínez-Caballero S. (2020). A Safe and Green Benzylic Radical Bromination Experiment. J. Chem. Educ..

[39] Ando K. (1999). A Mechanistic Study of the Horner–Wadsworth–Emmons Reaction: Computational Investigation on the Reaction Pass and the Stereochemistry in the Reaction of Lithium Enolate Derived from Trimethyl Phosphonoacetate with Acetaldehyde. J. Org. Chem..

[40] Ghiaci M., Asghari J. (1999). Dealkylation of Alkyl and Aryl Ethers with AlCl_3_—NaI in the Absence of Solvent. Synth. Commun..

[41] Samperna S., Zanotti C., Scafato P., Boari A., Visconti S., Vurro M., Superchi S., Evidente A., Marra M. (2023). (±)-3-Deoxyradicinin Induces Stomata Opening and Chloroplast Oxidative Stress in Tomato (*Solanum lycopersicum* L.). Int. J. Mol. Sci..

[42] Li C., Cheng B., Fang S., Zhou H., Gu Q., Xu J. (2018). Design, Syntheses and Lipid Accumulation Inhibitory Activities of Novel Resveratrol Mimics. Eur. J. Med. Chem..

[43] Wiley R.H., Jarboe C.H. (1956). 2-Pyrones. XVII. Aryl Hydrazones of Triacetic Lactone and Their Rearrangement to 1-Aryl-3-Carboxy-6-Methyl-4-Pyridazones. The Methyl Ether of Triacetic Lactone. J. Am. Chem. Soc..

[44] Rizzo A., Mayer R.J., Trauner D. (2019). Biomimetic Approach Toward Enterocin and Deoxyenterocin. J. Org. Chem..

