# Supplementary material for: Efficient Synthesis of the Fungal Phytotoxin (±)-3-Deoxyradicinin
Source: Toxins (Basel). 2026 Sep 24;18(10):412. doi: 10.3390/toxins18100412 (PMC13709763; doi:10.3390/toxins18100412)
Supplement: Supplementary file 1 [file toxins-18-00412-s001.zip › toxins-4529105-supplementary.pdf]

Supplementary Materials: Efficient Synthesis of the Fungal Phytotoxin (±)-3-Deoxyradicinin

Marilena Caporale, Giulia Marsico, Patrizia Scafato and Stefano Superchi

Table S1. Conditions for screening the direct side-chain elongation of compound **4**

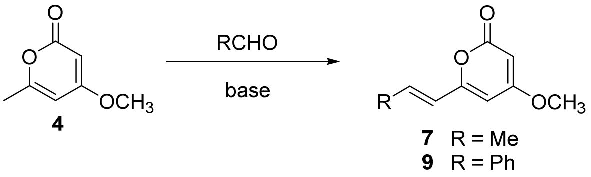

entry	aldehyde	base	reaction conditions	Product (Yield%)
1	MeCHO	$Mg(MeO)_2$	DMF, reflux, 6h	/
2	MeCHO	NaHMDS	THF, 0 °C, 2-3 h	/
3	MeCHO	MeONa	DMF, 0 °C, 2-3h	/
4	MeCHO	*t*-BuONa	DMF, RT, 30 min	**7** (traces)[a]
5	PhCHO	MeONa	DMF, RT, 1 h	**9** (27%)[b]
6	PhCHO	*t*-BuONa	DMF, RT, 30 min	**9** (57%)[b]

[a]Determined by ^{1}H-NMR of crude. [b]Isolated yield after column chromatography.

Table S2. Conditions for screening the benzylic bromination of **4**.[a]

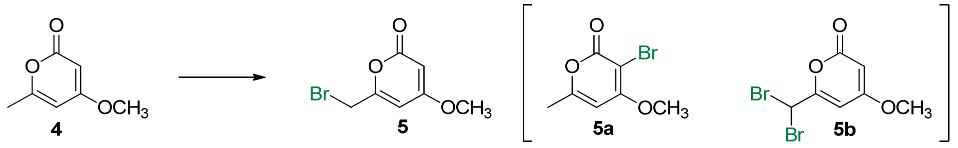

entry	brominating agent	solvent	T (°C)	t (h)	**4**:**5**:**5a**:**5b** ratio (%)[b]
1	*N*-bromosaccharin	EA	RT	1	0:0:100:0
2	*N*-bromosaccharin	EA	0°C	2	0:0:100:0
3	*N*-bromosaccharin	DCM	RT	1.5	0:0:100:0
4	*N*-bromosaccharin, BPO	DCM	0°C	0.5	0:0:100:0
5	*N*-bromosaccharin	CCl_4	0°C	0.5	0:0:100:0
6	TBCA	EA	reflux	6	100:0:0:0
7	TBCA	DCM	reflux	6	100:0:0:0
8[c]	NBS, h	CCl_4	reflux	1	0:80:12:8
9[d]	NBS, h	CCl_4	reflux	1	0:86:10:4
10	NBS, h	CCl_4	reflux	1	0:92[e]:5:3
11	NBS, BPO	CCl_4	reflux	3	0:90:7:3
12	NBS, AIBN	DMC	65-70°C	1	0:0:100:0
13	NBS, AIBN	MA	reflux	2	20:0:80:0
14	NBS, AIBN	ACN	65-70°C	1	0:0:100:0
15	NBS, AIBN	MTBE	reflux	24	25:0:75:0
16	NBS, AIBN	Water	65-70°C	24	40:0:60:0
17[f]	NBS	solid	RT	1	100:0:0:0

[a]Reaction conditions: substrate concentration 0.04 *M*, brominating agent 1.1 equiv., radical initiator 0.1 equiv. [b]Determined by GC-MS and ^{1}H-NMR (**5a**: Marrison, L.R. *et al.* *Bioorg. Med. Chem. Lett.* **2003**, *13*, 2667; **5b**: Bacardit, R. *et al.* *J. Heterocycl. Chem.* **1982**, *19*, 157) of the crude reaction mixture. [c]Substrate concentration 0.1 *M*. [d]Substrate concentration 0.08 *M*. [f]Solvent-free procedure carried out by grinding solid **4** and NBS in a mortar. [e]66% isolated yield. Ethyl acetate (EA); dichloromethane (DCM); dimethyl carbonate (DMC); methyl acetate (MA); acetonitrile (ACN); methyl *tert*-butyl ether (MTBE); benzoyl peroxide (BPO); 2,2'-azobis(2 methylpropionitrile) (AIBN).

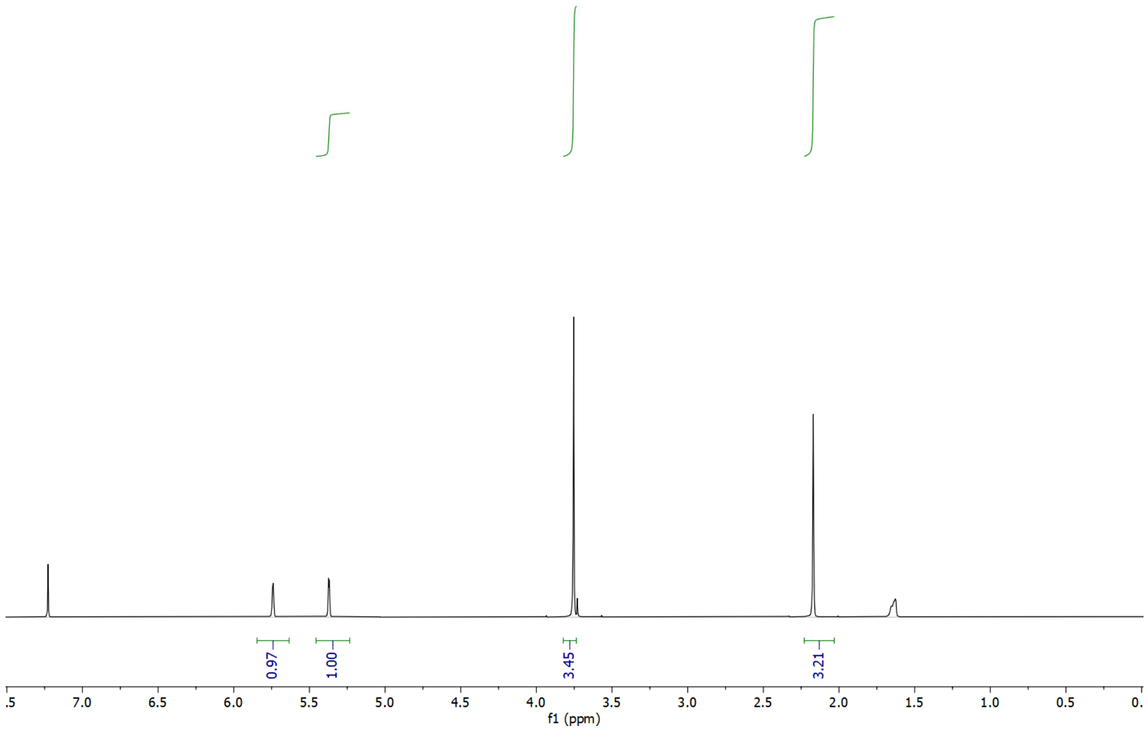

Figure S1. ^{1}H-NMR spectrum (400 MHz, $CDCl_3$) of 4-methoxy-6-methyl-2*H*-pyran-2-one (**4**).

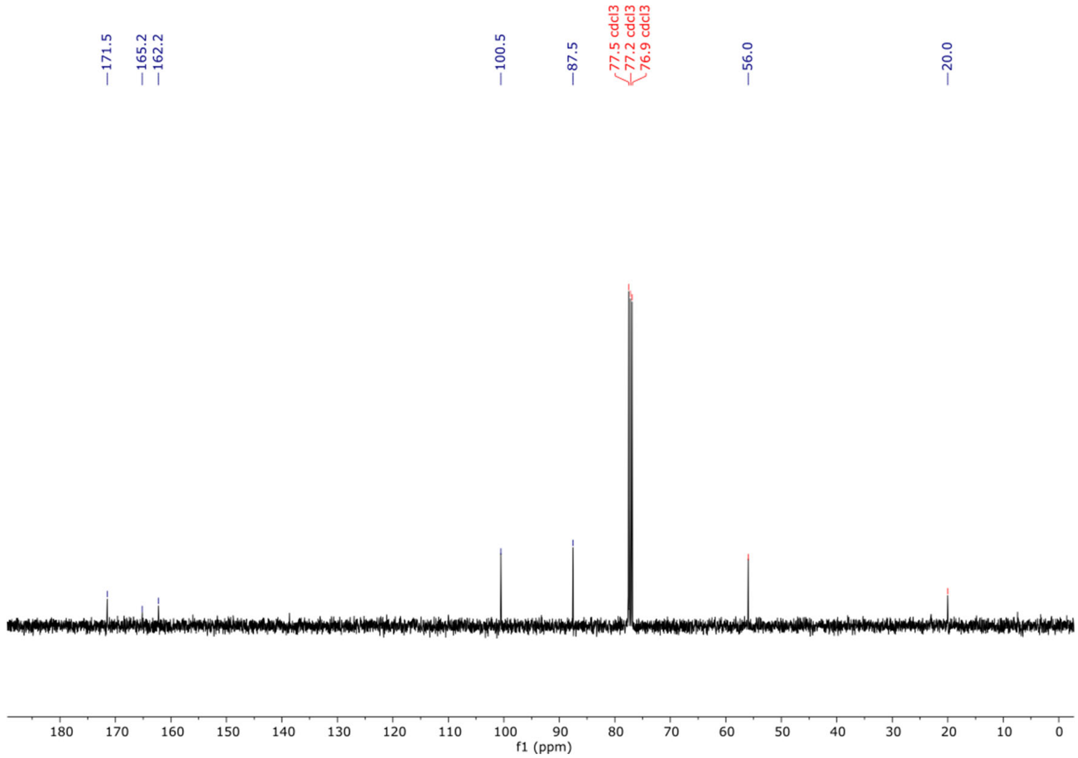

Figure S2. ^{13}C-NMR spectrum (100 MHz, $CDCl_3$) of 4-methoxy-6-methyl-2*H*-pyran-2-one (**4**).

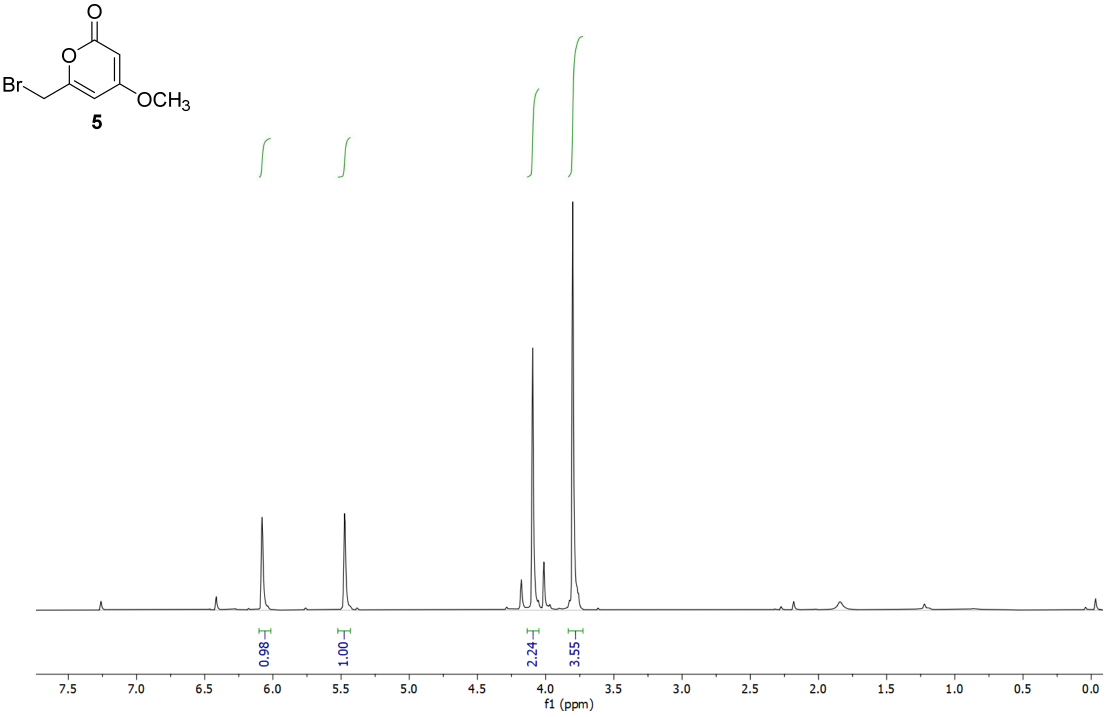

Figure S3. ^{1}H-NMR spectrum (400 MHz, $CDCl_3$) of 6-(bromomethyl)-4-methoxy-2*H*-pyran-2-one (**5**).

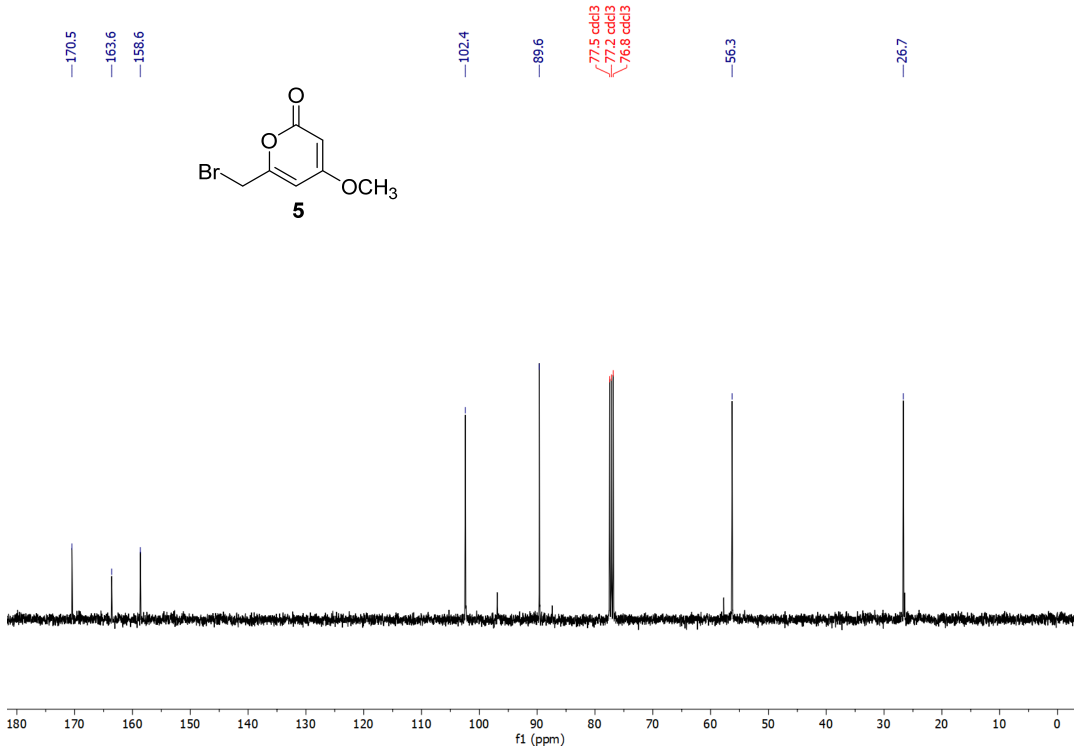

Figure S4. ^{13}C-NMR spectrum (100 MHz, $CDCl_3$) of 6-(bromomethyl)-4-methoxy-2*H*-pyran-2-one (**5**).

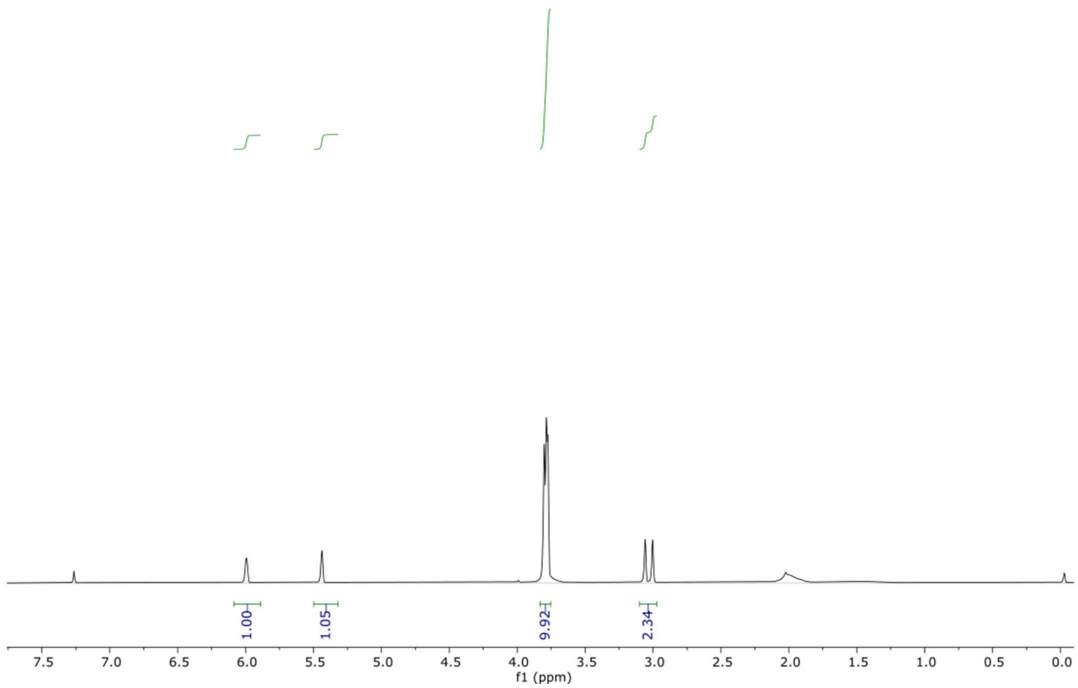

Figure S5. ^{1}H-NMR spectrum (400 MHz, $CDCl_3$) of dimethyl ((4-methoxy-2-oxo-2*H*-pyran-6-yl)methyl)phosphonate (**6**).

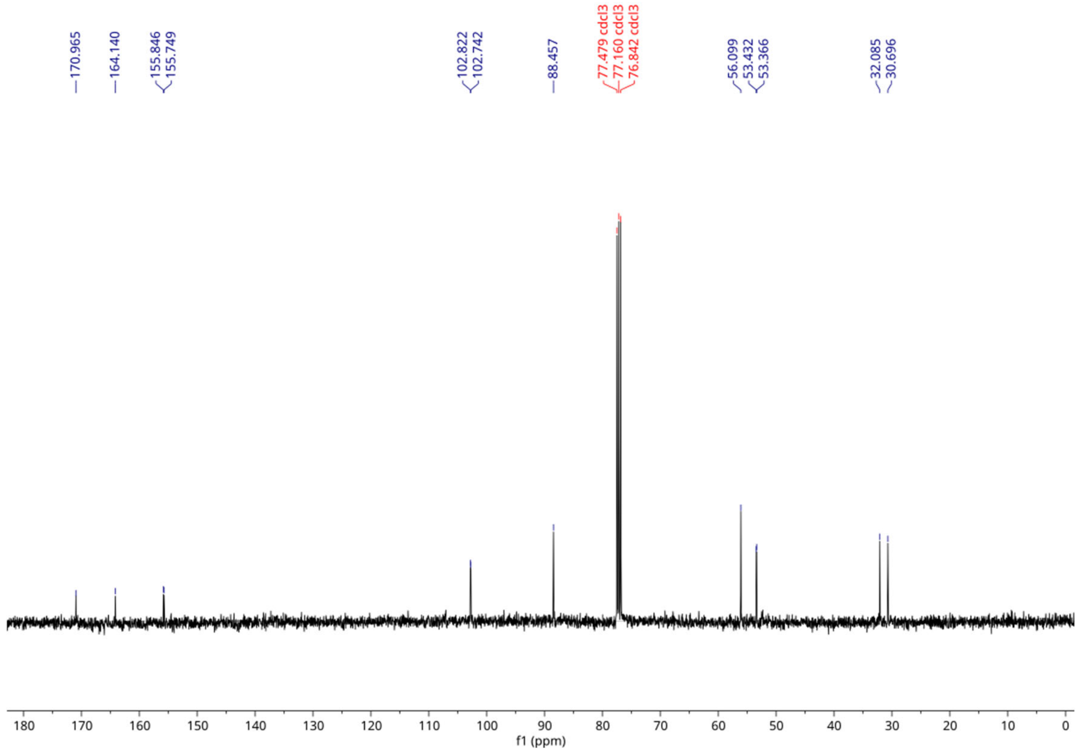

Figure S6. ^{13}C-NMR spectrum (100 MHz, $CDCl_3$) of dimethyl ((4-methoxy-2-oxo-2*H*-pyran-6-yl)methyl)phosphonate (**6**).

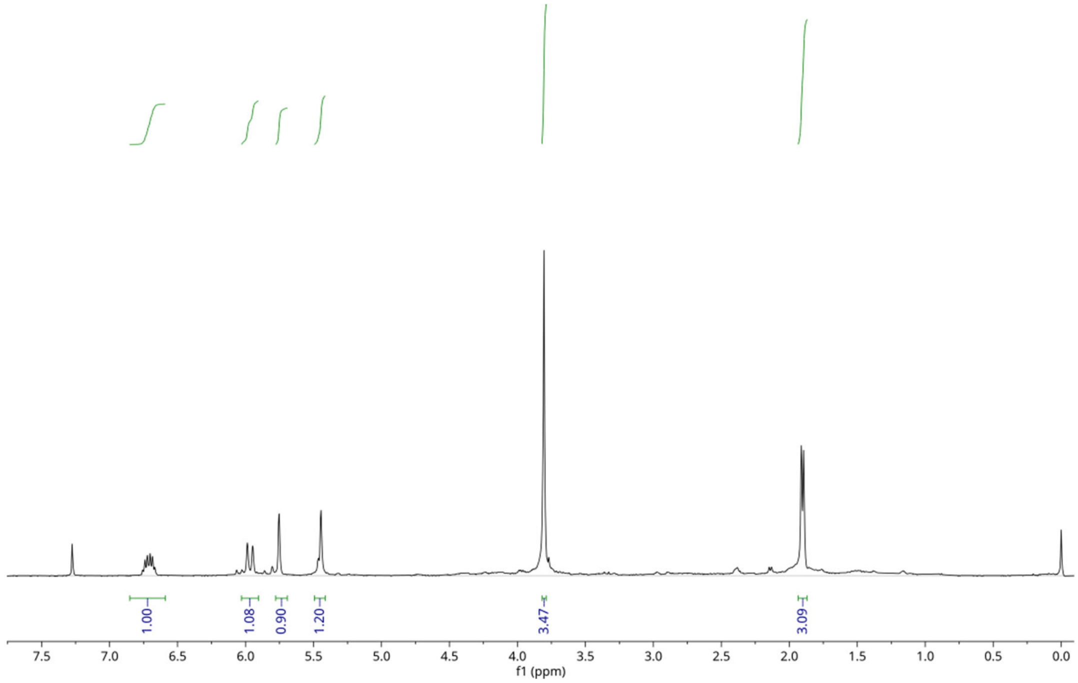

Figure S7. ^{1}H-NMR spectrum (400 MHz, $CDCl_3$) of (*E*)-4-methoxy-6-(prop-1-en-1-yl)-2*H*-pyran-2-one (**7**).

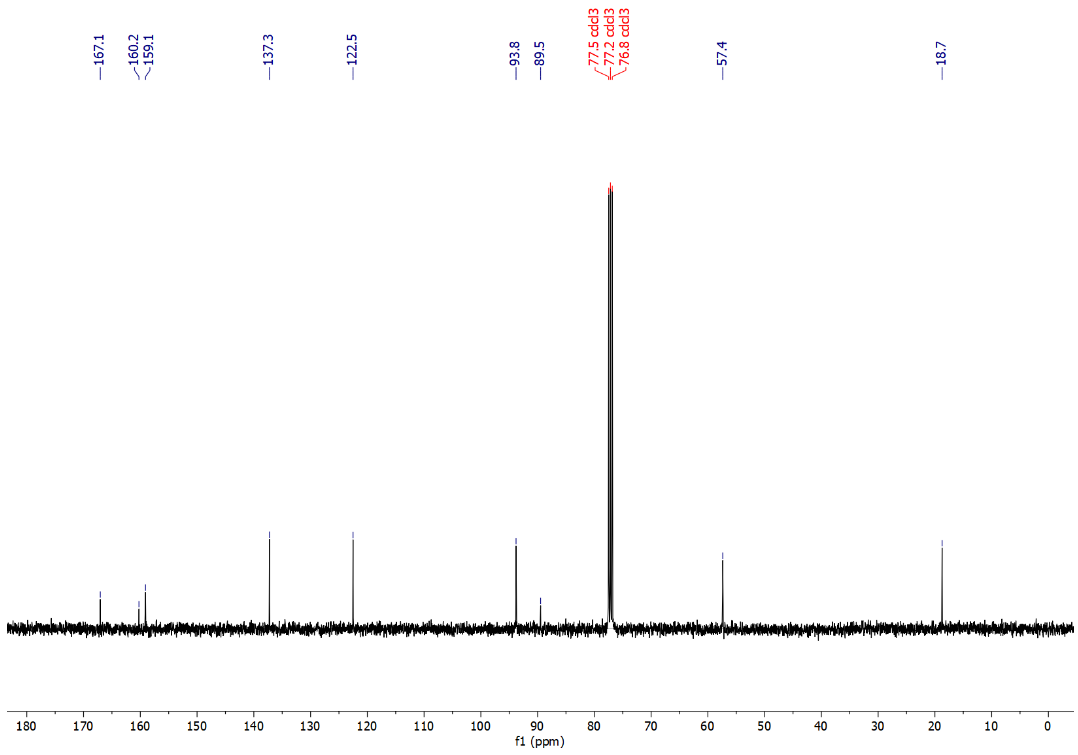

Figure S8. ^{13}C-NMR spectrum (100 MHz, $CDCl_3$) of (*E*)-4-methoxy-6-(prop-1-en-1-yl)-2*H*-pyran-2-one (**7**).

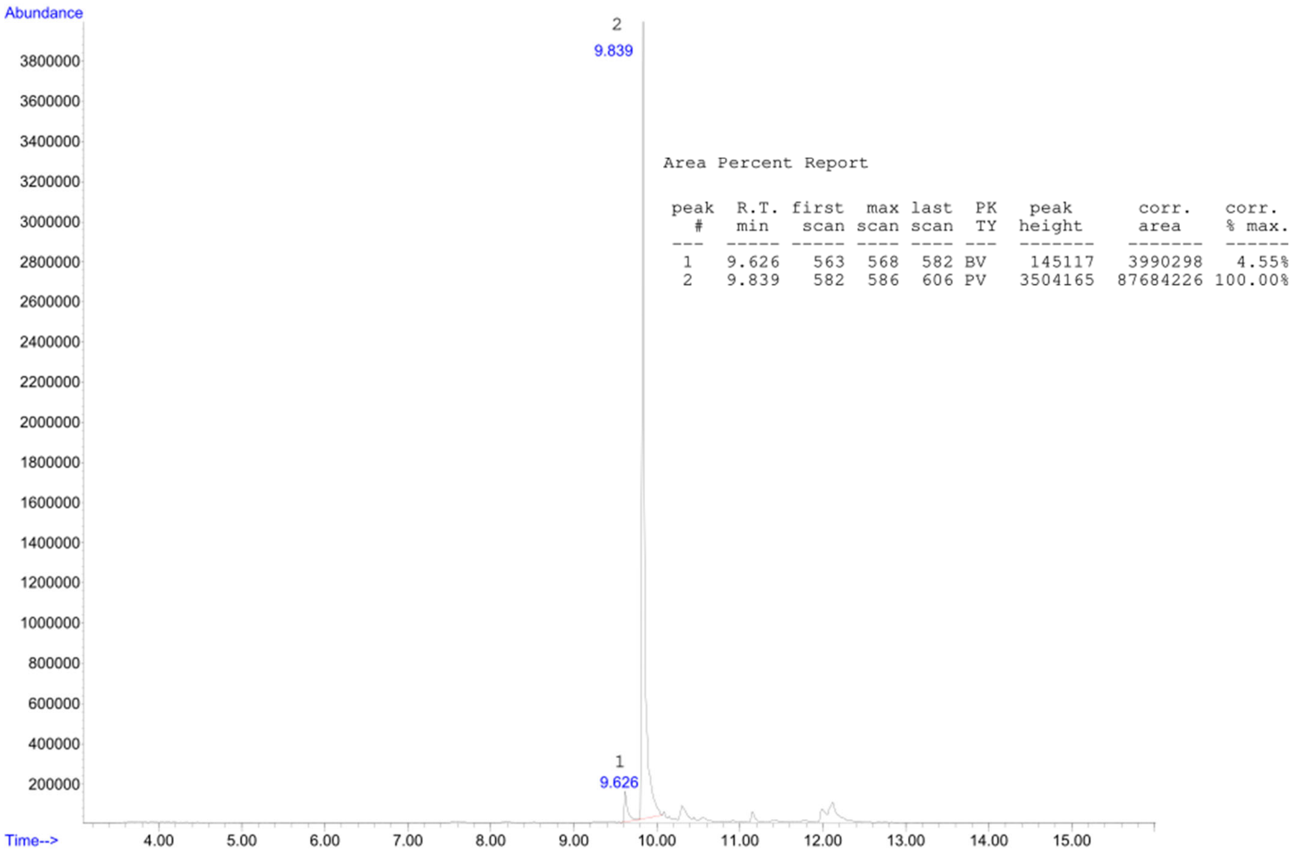

Figure S9. GC-MS chromatogram of olefin **7**. Peak 1 corresponds to the *Z* isomer, while peak 2 corresponds to the *E* isomer. The *E:Z* ratio was determined to be 96:4.

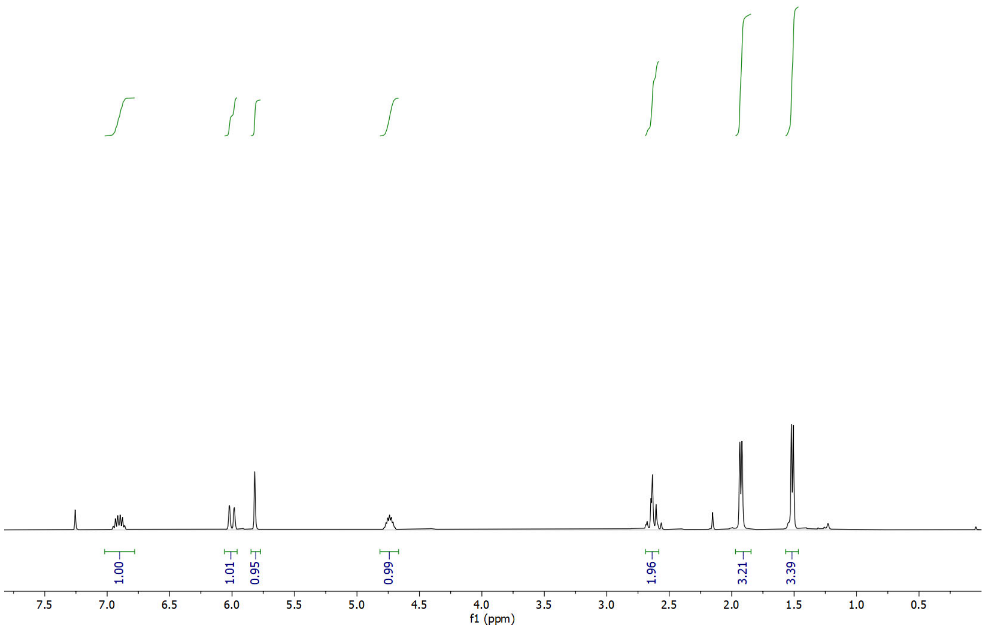

Figure S10. ^{1}H-NMR spectrum (400 MHz, $CDCl_3$) of (±)-3-deoxyradicinin (**2**).

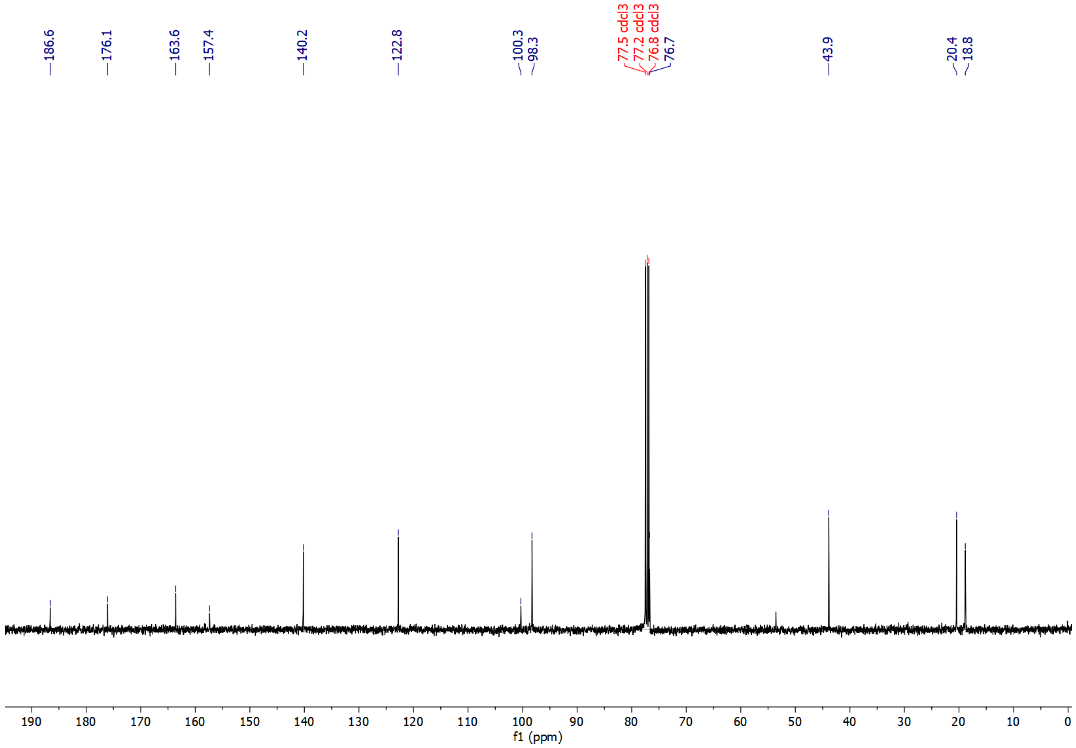

Figure S11. ^{13}C-NMR spectrum (100 MHz, $CDCl_3$) of (±)-3-deoxyradicinin (**2**).

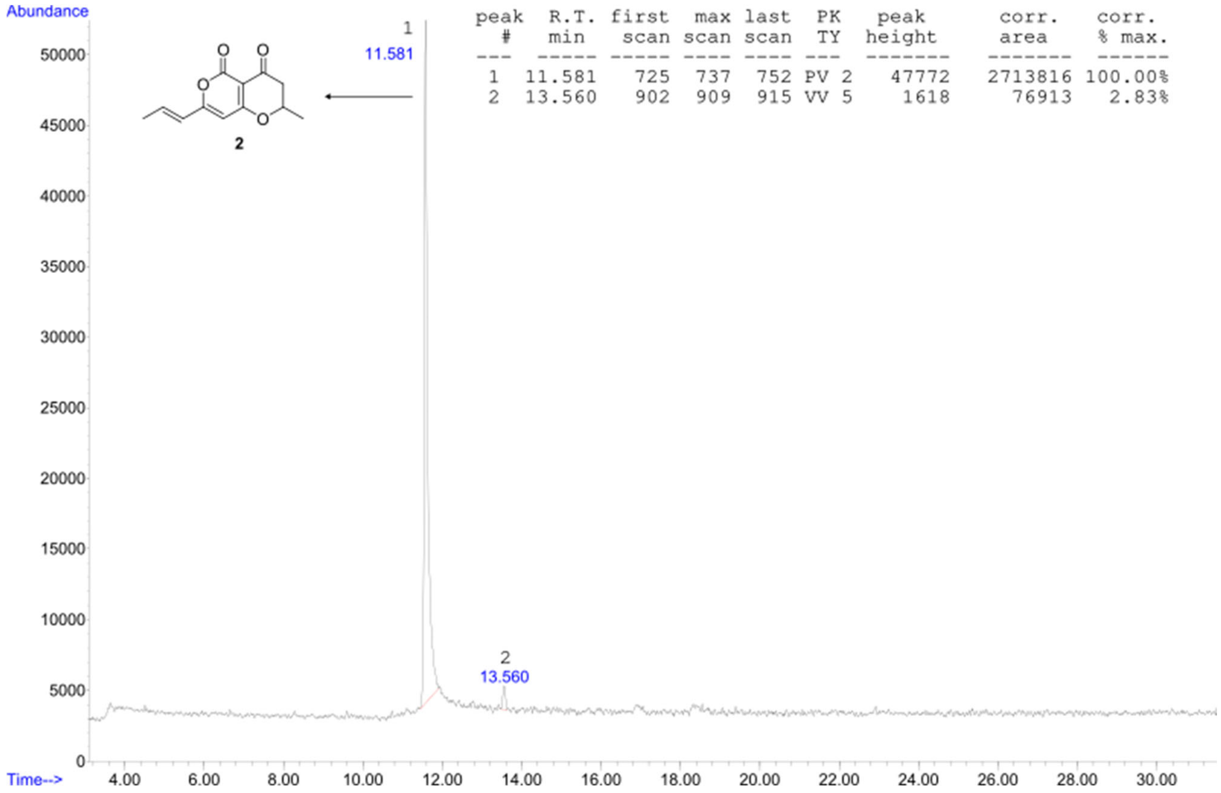

Figure S12. GC-MS chromatogram of (±)-3-deoxyradicinin (**2**).

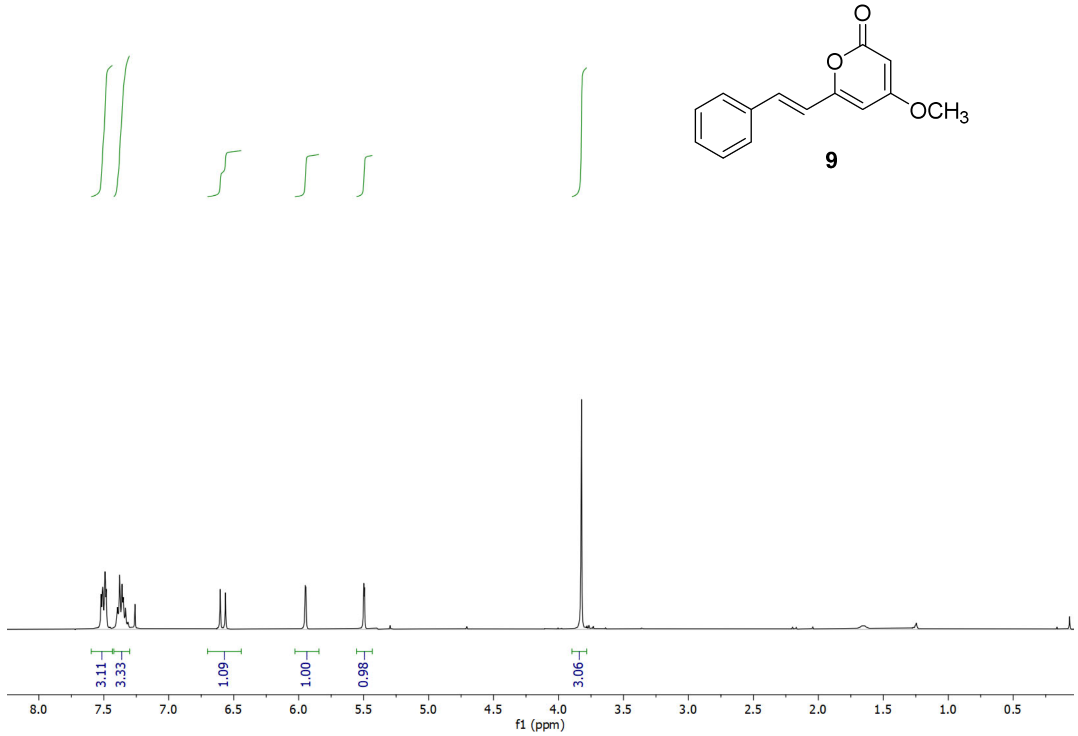

Figure S13. ^{1}H-NMR spectrum (400 MHz, $CDCl_3$) of 5,6-dehydrokawain (**9**).

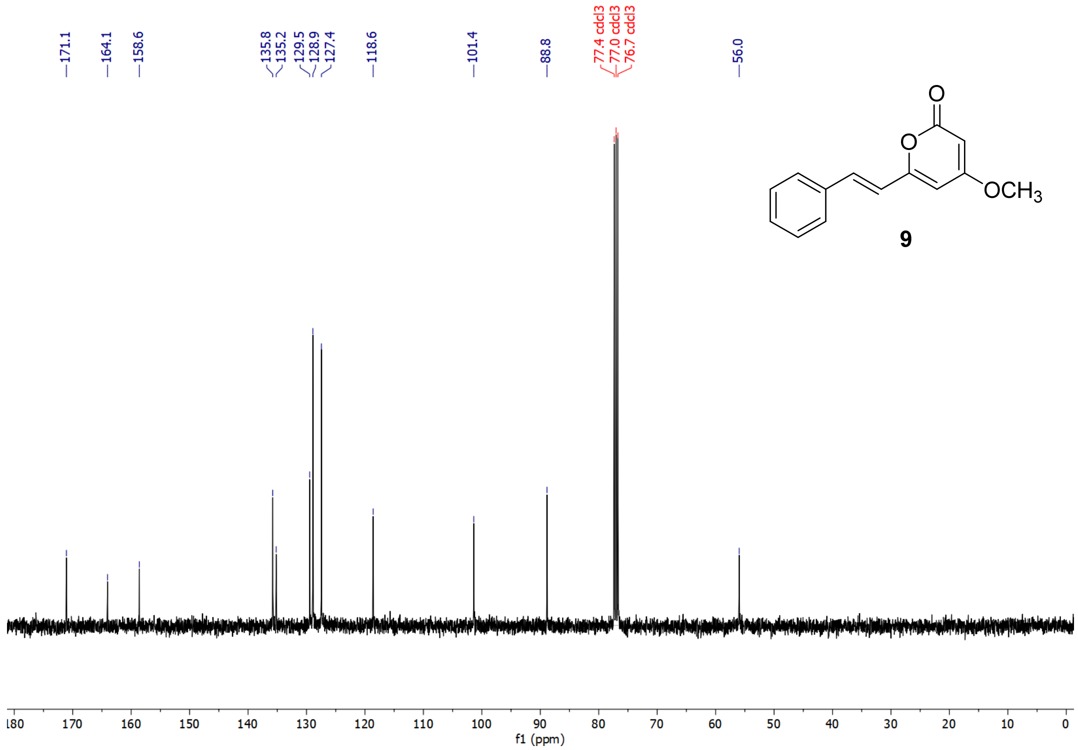

Figure S14. ^{13}C-NMR spectrum (100 MHz, $CDCl_3$) of 5,6-dehydrokawain (**9**).
